# Organization of the core respiratory network: Insights from optogenetic and modeling studies

**DOI:** 10.1371/journal.pcbi.1006148

**Published:** 2018-04-26

**Authors:** Jessica Ausborn, Hidehiko Koizumi, William H. Barnett, Tibin T. John, Ruli Zhang, Yaroslav I. Molkov, Jeffrey C. Smith, Ilya A. Rybak

**Affiliations:** 1 Department of Neurobiology and Anatomy, Drexel University College of Medicine, Philadelphia, United States of America; 2 Cellular and Systems Neurobiology Section, National Institute of Neurological Disorders and Stroke, National Institutes of Health, Bethesda, United States of America; 3 Department of Mathematics and Statistics, Georgia State University, Atlanta, United States of America; Northeastern University, UNITED STATES

## Abstract

The circuit organization within the mammalian brainstem respiratory network, specifically within and between the pre-Bötzinger (pre-BötC) and Bötzinger (BötC) complexes, and the roles of these circuits in respiratory pattern generation are continuously debated. We address these issues with a combination of optogenetic experiments and modeling studies. We used transgenic mice expressing channelrhodopsin-2 under the VGAT-promoter to investigate perturbations of respiratory circuit activity by site-specific photostimulation of inhibitory neurons within the pre-BötC or BötC. The stimulation effects were dependent on the intensity and phase of the photostimulation. Specifically: (1) Low intensity (≤ 1.0 mW) pulses delivered to the pre-BötC during inspiration did not terminate activity, whereas stronger stimulations (≥ 2.0 mW) terminated inspiration. (2) When the pre-BötC stimulation ended in or was applied during expiration, rebound activation of inspiration occurred after a fixed latency. (3) Relatively weak sustained stimulation (20 Hz, 0.5–2.0 mW) of pre-BötC inhibitory neurons increased respiratory frequency, while a further increase of stimulus intensity (> 3.0 mW) reduced frequency and finally (≥ 5.0 mW) terminated respiratory oscillations. (4) Single pulses (0.2–5.0 s) applied to the BötC inhibited rhythmic activity for the duration of the stimulation. (5) Sustained stimulation (20 Hz, 0.5–3.0 mW) of the BötC reduced respiratory frequency and finally led to apnea. We have revised our computational model of pre-BötC and BötC microcircuits by incorporating an additional population of post-inspiratory inhibitory neurons in the pre-BötC that interacts with other neurons in the network. This model was able to reproduce the above experimental findings as well as previously published results of optogenetic activation of pre-BötC or BötC neurons obtained by other laboratories. The proposed organization of pre-BötC and BötC circuits leads to testable predictions about their specific roles in respiratory pattern generation and provides important insights into key circuit interactions operating within brainstem respiratory networks.

## Author summary

We investigated the organization of key circuits within the brainstem respiratory central pattern generator (CPG) responsible for generating the basic breathing pattern in mammals. Our study focused on the organization and interactions of two core components of this CPG: the pre-Bötzinger (pre-BötC) and Bötzinger (BötC) complexes. We used genetically transformed mice with inhibitory neurons containing light activated membrane channels, allowing us to investigate perturbations of respiratory activity during site-specific activation of pre-BötC or BötC inhibitory neurons by applying laser photostimuli of different intensities and durations at different phases of the respiratory cycle. Photostimulation of inhibitory neurons caused major disturbances of circuit activity. The obtained experimental results were used to extend and refine a computational model simulating circuit interactions within and between the pre-BötC and BötC. A major feature of the model is a novel inhibitory post-inspiratory population within the pre-BötC and its interactions with other populations in the network. We validated the model by successfully testing its ability to reproduce our experimental results as well as results of previous studies that employed photoactivation of pre-BötC or BötC neurons. Our study provides important insights into the circuit organization in the brainstem respiratory CPG with implications for other circuits controlling rhythmic behaviors.

## Introduction

Rhythmic movements such as breathing and locomotion are produced in the nervous system by central pattern generator (CPG) networks consisting of interacting inhibitory and excitatory circuits that constitute the basic neural machinery generating motor behavior. Defining the structural-functional arrangement of CPG circuits remains a central problem in many neural motor control systems. In the present studies, we have addressed this problem of delineating the configuration of core circuits of the mammalian brainstem respiratory CPG.

The basic pattern of respiratory activity includes three major phases: inspiration, post-inspiration, and late expiration [1,2]. This pattern is generated within bilaterally located medullary ventral respiratory columns (VRC), each of which includes two key compartments, the pre-Bötzinger (pre-BötC) and Bötzinger (BötC) complexes, postulated to represent the core of the respiratory CPG [3–11]. The pre-BötC contains a heterogeneous population of excitatory neurons, including cells with intrinsic bursting properties, with mutual synaptic interconnections that generate rhythmic bursts of inspiratory activity and drive inspiratory motor output [12–17]. This structure also contains circuits of GABAergic and glycinergic inhibitory neurons [18–21], which are active during the inspiratory phase and provide phasic inhibition that is widely projected to other brainstem compartments, including to BötC circuits to inhibit their expiratory neurons. The BötC mostly contains post-inspiratory and augmenting expiratory neurons that provide post-inspiratory and expiratory inhibition [22–25]. These neurons inhibit pre-BötC inspiratory neurons during expiration to coordinate expiratory and inspiratory phases of breathing. Although post-inspiratory neurons are predominantly located in the BötC, they are widely distributed within the VRC and present in other brainstem compartments including the pre-BötC [9,26,27].

The current experimental data suggest that generation and shaping of the basic respiratory pattern are based on the intrinsic bursting properties of, and interactions between, the excitatory neurons within the pre-BötC and involve inhibitory interactions between different neuron populations within and between the pre-BötC and BötC regions. The pre-BötC/BötC core circuitry is proposed to interact with other brainstem compartments, including several pontine structures [3,28–30], the retrotrapezoid nucleus/parafacial respiratory group (RTN/pFRG; [31–36], and the nucleus tractus solitarii (NTS, mediating multiple inputs and feedbacks to the respiratory network), which modulate and adjust operation of the core respiratory network and the pattern of generated respiratory activity to the current physiological and metabolic conditions [37–39].

The basic connectome of interactions among respiratory neuron populations in the pre-BötC and BötC compartments has been proposed in a series of computational models [3–8,10,11,40]. These models followed the key conceptual suggestions that (1) although the pre-BötC is capable of autonomous generation of rhythmic bursting when isolated *in vitro*, in more intact preparations and *in vivo* this structure is embedded in a larger respiratory network where its operation is modulated by interactions with other brainstem compartments, and particularly depends on mutual inhibitory interactions with the BötC, so that both the pre-BötC and BötC are critically involved in the generation and shaping of the normal (eupneic) three-phase rhythmic respiratory pattern, and (2) inhibitory interactions between and within these core compartments are fundamentally involved in generating the normal rhythmic respiratory neural activity. Accordingly, disruption of these interactions may cause a switch from the eupneic neural pattern of breathing defined by network interactions to either an intrinsic rhythmic activity originating within the pre-BötC, or to apnea or an abnormal apneustic activity.

The above concept and the related computational models were indirectly supported by multiple experimental data [2,41–44]. However, this concept and, specifically, the critical role of inhibitory interactions in respiratory rhythm generation were challenged by a study from Janczewski et al. [45], in which fast inhibitory neurotransmission was pharmacologically suppressed in anesthetized rats *in vivo*. Although these authors acknowledged the important role of inhibition in “shaping the pattern of respiratory motor output, assuring its stability, and in mediating reflex or volitional apnea”, they concluded that: (1) “postsynaptic inhibition within the pre-BötC and BötC is not essential for generation of normal respiratory rhythm in intact mammals” [45], and (2) “bilateral ablation of BötC did not result in apnea” and did not change respiratory frequency and phase durations [45] and hence the BötC does not play a role in respiratory rhythm generation during normal eupneic breathing. Subsequently Sherman et al. [46], who optogenetically targeted glycinergic pre-BötC neurons in mice to investigate their role in respiratory rhythm generation, similarly concluded that local inhibitory neurons are not essential for rhythm generation. However, the latter study was limited to optogenetic manipulation of only glycinergic neurons in the pre-BötC and did not investigate the potential role of the BötC and its interactions with the pre-BötC. In contrast, another recent study [47] showed that (a) optogenetic activation of BötC neurons resulted in depression of the respiratory rhythm, and (b) the effects of transient optogenetic stimulation of pre-BötC neurons depended on the phase of the respiratory cycle at which it was applied. Specifically, there was an insensitive post-inspiratory period during which the next inspiratory burst could not be triggered by optogenetic stimulation of the pre-BötC [47]. This insensitive period could result from a post-inspiratory inhibition originating in the BötC or within the pre-BötC. The results of these studies are consistent with the original concept of an important contribution of BötC inhibitory circuits and their interactions with pre-BötC circuits in respiratory rhythm and pattern generation. Resolving these issues requires systematic investigations using site-specific targeting of inhibitory neurons in the pre-BötC and BötC.

In the present study, we thus employed an optogenetic strategy with a transgenic mouse line expressing Cre recombinase controlled by the vesicular GABA transporter (VGAT) promoter to express Channelrhodopsin-2 (ChR2) in both GABAergic and glycinergic inhibitory neurons. Our experiments were performed with *in situ* perfused brainstem-spinal cord preparations from adult transgenic mice that readily enable site-specific laser stimulation of inhibitory neurons within the pre-BötC and BötC regions during electrophysiological recording for analysis of perturbations of circuit activity and motor output. Single laser pulses of various intensities delivered at different phases of the respiratory cycle as well as series of pulses or sustained laser stimulations were applied either to the pre-BötC or the BötC regions. The photo-induced perturbations were regionally-specific and were dependent on stimulus intensity, duration, and phase of photostimulation in the respiratory cycle. The results of these studies were consistent with our original concept confirming an important role of the BötC and of inhibitory circuit interactions between the pre-BötC and the BötC for operation of the respiratory CPG.

To further examine and explain the results obtained in our study we revised our previous computational model of pre-BötC and BötC microcircuits by incorporating an additional population of post-inspiratory inhibitory neurons (post-I) in the pre-BötC which was based on the knowledge that these neurons are widely distributed in the VRC, including the pre-BötC [1,2,9,27,48]. The revised model was able to reproduce our experimental perturbations of circuit activity with sustained and phase-dependent short-duration activation of inhibitory neurons in the pre-BötC and BötC. The model was then further tested by its ability to reproduce previously published data showing the effects of site-specific photostimulation of neurons in the pre-BötC and BötC regions [47], which were reproduced without any additional adjustments to the model parameters. The circuit configuration proposed in the model leads to several testable predictions about respiratory network organization and the specific roles of different brainstem neuron populations and how their interactions contribute to generating and shaping the neural breathing pattern.

## Results

We utilized a combination of experimental and modeling studies to investigate inhibitory circuit interactions within and between the pre-Bötzinger (pre-BötC) and Bötzinger (BötC) complexes representing a core of the brainstem respiratory network responsible for generating and shaping the basic rhythmic pattern of respiratory activity in mammals. We used transgenic mice expressing channelrhodopsin-2 controlled by Cre recombinase expression driven by the VGAT promoter to investigate the effects of site-specific activation of inhibitory neurons in the pre-BötC or BötC by laser stimuli of different intensities and durations at different phases of the respiratory cycle. The results were used to extend our computational model simulating circuit interactions within and between the pre-BötC and BötC regions. The updated model was validated by testing its ability to reproduce the results of our experiments as well as the results of previous studies that employed optogenetic activation of pre-BötC and/or BötC neurons.

### Cre-dependent targeting of inhibitory neurons in the pre-BötC and BötC regions

**Fig 1:**
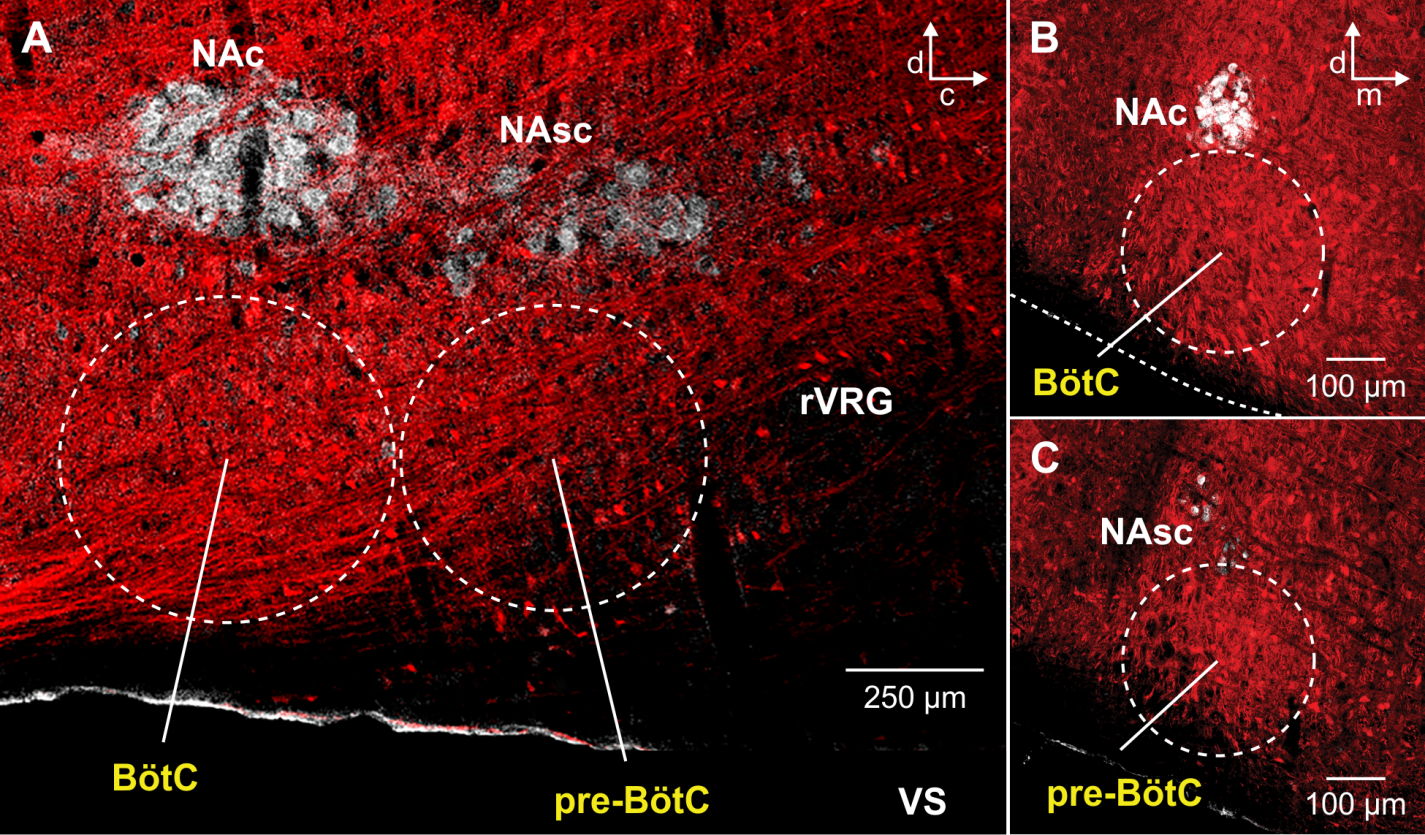
Spatial distribution of Cre-dependent tdTomato-labeled neurons in the medullary reticular formation in VGAT-tdTomato double Tg mouse strain. (**A**) Confocal fluorescence microscopy images of parasagittal sections at the level of NA (immunostained with ChAT antibody, white). (**B** and **C**) Coronal sections at the level of the BötC (B) and the pre-BötC (C) showing extensive distribution of VGAT-Cre driven tdTomato labeled neuronal somas and processes (red) throughout the ventral medullary reticular formation in an adult VGAT-tdTomato mouse. Abbreviations: pre-BötC, pre-Bötzinger complex; BötC, Bötzinger complex; rVRG, rostral ventrolateral respiratory group, NAc and NAsc, nucleus ambiguus (NA) compact and semi-compact subdivisions; VS, ventral surface; d, dorsal; c, caudal; m, medial.

**Fig 2:**
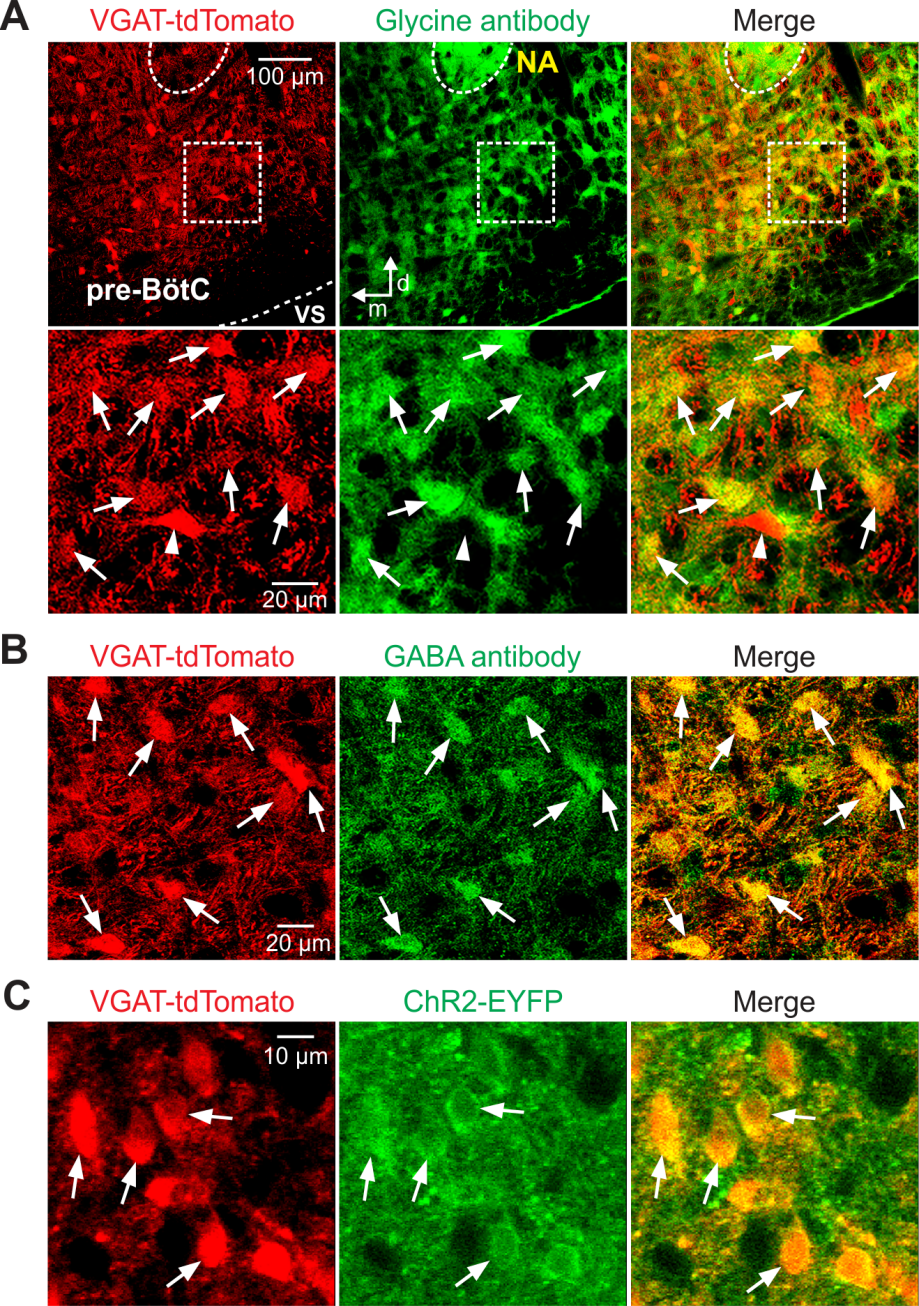
Glycine and GABA antibody, and ChR2-EYFP expression in Cre-dependent tdTomato-labeled neurons in the pre-BötC region in VGAT-tdTomato or VGAT-tdTomato-ChR2-EYFP mouse strains. (**A**) Confocal fluorescence microscopy images of the pre-BötC region at low magnification (upper panels) and a pre-BötC subregion at higher magnification (lower panels) in a representative fixed coronal section from an adult VGAT-tdTomato mouse showing VGAT-Cre dependent tdTomato-labeled neurons (red) and glycine antibody labeling of neurons (green), and co-localization (arrows) of glycine antibody labeling in VGAT-expressing pre-BötC neurons in merged images (Yellow). Example of VGAT-expressing tdTomato-labeled neuron without glycine antibody expression (arrowhead) in this histological section is also indicated. (**B**) Confocal fluorescence microscopy images of a subregion within the pre-BötC in a fixed coronal section from the VGAT-tdTomato mouse line, showing VGAT-Cre dependent tdTomato labeling (red) and GABA antibody labeling (green) in pre-BötC neurons. Merged image illustrates co-localization of GABA antibody labeling in tdTomato-labeled neurons. Examples of tdTomato-labeled neurons without GABA antibody expression (arrowheads) are also indicated. (**C**) Two-photon microscopy single optical plane “live” images of the pre-BötC subregion in an *in vitro* neonatal medullary slice from the VGAT-tdTomato-ChR2-EYFP mouse line, illustrating that tdTomato-labeled VGAT-positive pre-BötC neurons (red) express ChR2-EYFP (green) in somal membranes, as confirmed in the merged image. All images have the same dorso-medial anatomical orientation. Abbreviations: d, dorsal; m, medial; NA, nucleus ambiguus; VS, ventral surface.

We histologically validated the VGAT-Cre driver mouse line by verifying, via confocal fluorescence microscopy in fixed serial sections, Cre-driven tdTomato labeling of neurons in the pre-BötC and BötC (Fig 1) in the double Tg line VGAT-tdTomato (n = 5), and by confirming co-labeling of glycine antibody (Fig 2A) or GABA antibody (Fig 2B) in tdTomato-expressing neurons within the pre-BötC and BötC in the VGAT-tdTomato line (n = 2 each). We also confirmed tdTomato labeling of neurons distributed extensively throughout the medullary reticular formation, including in ventrolateral medullary respiratory regions caudal and adjacent to the pre-BötC (e.g., in the rostral ventrolateral respiratory group (rVRG) (Fig 1). We subsequently verified Cre-driven ChR2-EYFP expression in VGAT-positive tdTomato labeled neurons within the pre-BötC and BötC regions in the triple Tg line VGAT-tdTomato-ChR2-EYFP (n = 3) by two-photon microscopy in “live” neonatal medullary slices *in vitro*. The ChR2-EYFP fusion protein was heavily expressed in processes and somal membranes of VGAT-tdTomato labeled neurons within the pre-BötC (Fig 2C) and BötC.

### Optogenetic photostimulation of functionally identified ChR2-expressing pre-BötC inspiratory VGAT-positive neurons *in vitro*

**Fig 3:**
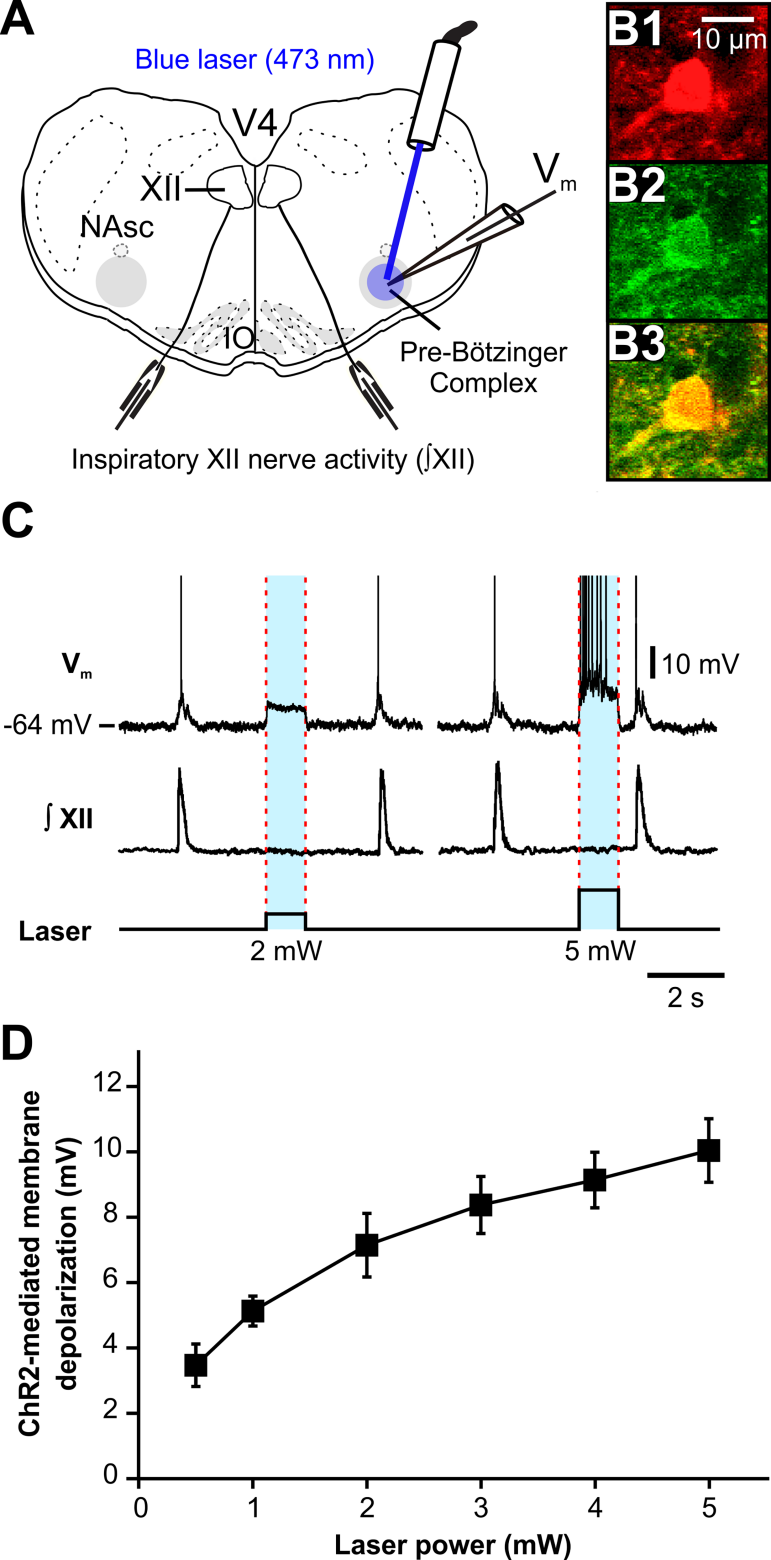
Photostimulation causes membrane depolarization of rhythmically active ChR2-expressing pre-BötC inspiratory VGAT-positive neurons *in vitro*. **(A)** Schematic of the *in vitro* rhythmic slice preparation from a neonatal VGAT-ChR2 transgenic mouse illustrating whole-cell current-clamp recordings from pre-BötC inspiratory VGAT-positive neurons with unilateral pre-BötC laser illumination (0.5–5 mW) and suction-electrode recordings from hypoglossal (XII) nerves to monitor inspiratory activity. NAsc, semi-compact division of nucleus ambiguus; V4, fourth ventricle; IO, inferior olivary nucleus. The pre-BötC regions are indicated by gray circles (~300 μm diameter). (**B)** Two-photon single-optical plane images of a pre-BötC inspiratory neuron targeted for whole-cell recording, showing VGAT-Cre driven tdTomato labeling (**B1**), ChR2-EYFP expression (**B2**) and confirmed co-expression in merged image (**B3**). (**C**) Current-clamp recording from a VGAT-positive inhibitory pre-BötC inspiratory neuron in **B** illustrating inspiratory spikes synchronized with integrated inspiratory XII nerve activity (∫XII). The membrane potential (*V*m) of this neuron was depolarized by ~7 mV at 2 mW and by ~10 mV at 5 mW of laser power (spikes are truncated). The neuron was hyperpolarized from resting baseline potential to -64 mV by applied constant current in this example to reveal the magnitude of the light-induced membrane depolarization. The lower trace indicates the duration and amplitude of the laser stimulation. (**D**) Summary data (n = 8 neurons from 3 slice preparations, mean ± SEM) showing that ChR2-mediated membrane depolarization of VGAT-positive pre-BötC inspiratory neurons was laser power-dependent.

To verify the efficacy of photostimulation (473 nm, 0.5–5 mW) of ChR2-expressing pre-BötC inspiratory VGAT-positive neurons, we performed combined optogenetic stimulation and whole-cell patch-clamp recordings in rhythmically active *in vitro* neonatal medullary slice preparations from the VGAT-tdTomato-ChR2-EYFP mouse lines (n = 3). These slices effectively isolate the bilateral pre-BötC along with hypoglossal (XII) motoneurons to monitor inspiratory XII activity [17], allowing whole-cell recordings from rhythmically active inspiratory pre-BötC neurons and simultaneous laser illumination to the recorded neuron (Fig 3*A*). We performed current-clamp recording from tdTomato-labeled neurons, in which co-expression of ChR2 was confirmed with two-photon single optical plane live images (Fig 3B), and identified rhythmically active pre-BötC inspiratory neurons, which were active in phase with integrated inspiratory XII nerve activity (∫XII). The membrane potential of these functionally identified VGAT-positive pre-BötC inspiratory neurons was depolarized by 10.0 ± 1.0 mV at 5 mW laser power (*n =* 8 neurons) with fast kinetics occurring within 12 ms, and recovery within 25 ms after terminating laser illumination (Fig 3*C*). Summary data (n = 8 neurons from 3 slice preparations, mean ± SEM) shown in Fig 3D illustrates that ChR2-mediated depolarization of VGAT-positive inspiratory pre-BötC neurons was laser power-dependent, and importantly demonstrates that laser illumination caused membrane depolarization of VGAT-positive (inhibitory) inspiratory neurons in all cases with the applied laser power ranging from 0.5 to 5 mW. The observed membrane depolarization of these neurons could also include inhibitory effects on these neurons due to photostimulation of ChR2-expressing terminals from other VGAT-positive neurons with somas located outside of the pre-BötC providing inhibitory synaptic connections to the VGAT-positive neurons within the pre-BötC. However, our results indicate that these possible inhibitory effects were small, and therefore the optogenetic experimental results obtained in our study in slices *in vitro* were always attributed to excitatory effects (i.e., membrane depolarization) on VGAT-positive inspiratory neurons within the pre-BötC (as in Fig 3C).

### Perturbations of the respiratory rhythm by short-duration photostimulation of VGAT-expressing inhibitory neurons within the pre-BötC

**Fig 4:**
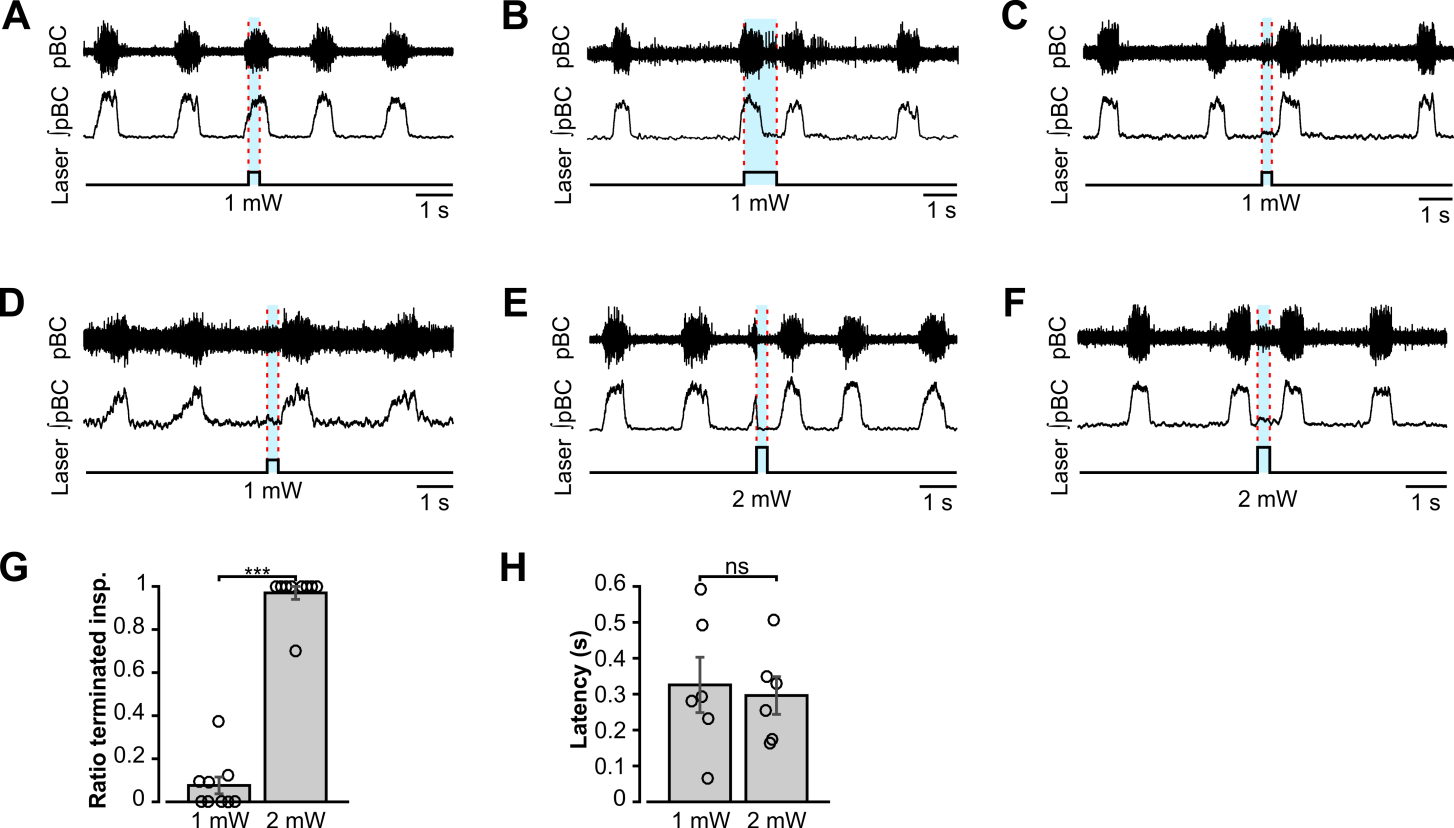
Perturbations of the respiratory rhythm by bilateral photostimulation of VGAT-expressing inhibitory neurons within the pre-Bötzinger complex *in situ*. (**A-F**) Representative examples of the effects of short light pulses of different intensity applied at different phases of the respiratory cycle. In each diagram, the upper traces show extracellular recordings from the pre-BötC (pBC) inspiratory activity, the middle traces show integrated pre-BötC activity (∫pBC), and the bottom traces show the laser stimulus application with the laser intensity given below the stimulus. The timing of the photostimulation is indicated in all three traces by blue shading and dashed red lines. (**A**) Low-intensity (1.0 mW) photostimulation (300 ms) applied during the inspiratory phase did not perturb the ongoing respiratory rhythm. (**B**) Same low-intensity laser stimulation (as in A) but with longer duration (1 s) to outlast the inspiratory phase did not terminate inspiration, but caused rebound excitation after ending the stimulus which led to an advanced onset of the next inspiratory phase. (**C**) Low-intensity (1.0 mW) photostimulation (300 ms) applied during the expiratory phase caused rebound excitation after the end of the stimulus leading to an advanced onset of the next inspiratory phase. (**D**) When the 300 ms, 1.0 mW laser pulse was applied at the end of the expiratory phase, inspiration was delayed for the duration of the stimulus and after the light stimulation was turned off. (**E**) High-intensity (2.0 mW) photostimulation (300 ms) applied during the inspiratory phase terminated inspiration and elicited delayed rebound excitation of inspiration after the stimulus ended. (**F**) The same 2.0 mW stimulation applied during the expiratory phase caused rebound excitation after the end of the stimulus leading to an advanced onset of the next inspiratory phase, similar to the examples shown in panels B, C and E. (**G**) Population data (n = 9, mean ± SEM, ***p ≤ 0.001) showing that the 2.0 mW stimulations reliably terminated inspiration while 1.0 mW stimulations did not. The open circles indicate the average number of terminated inspiratory bursts normalized to the total number of bursts for each animal. (**H**) The latency between the end of the light stimulus and the onset of the next inspiration was independent of photostimulation intensity (n = 6, mean ± SEM).

To investigate the role of inhibitory neurons within the pre-BötC in the generation and control of the respiratory pattern, we used site-specific optogenetic stimulations of VGAT expressing inhibitory neurons by single laser pulses of different durations (0.3–1.0 s) and power (1.0 or 2.0 mW) delivered bilaterally to both (left and right) pre-BötC regions at different phases of the respiratory cycle. The perturbations of respiratory neural activity induced by these photostimulations were dependent on the phase of application and the intensity of the applied laser pulses. Low intensity (1.0 mW) single pulses of 300 ms duration delivered to the pre-BötC during the inspiratory phase did not affect the respiratory rhythm (n = 9, Fig 4A and 4G). In contrast, the same low-intensity stimuli outlasting the inspiratory phase (n = 6, Fig 4B) or applied during the expiratory phase (n = 6, Fig 4C), induced rebound activation and thus an advanced onset of the next inspiratory phase. Only when the stimulus occurred very late during the expiratory phase and lasted into the next inspiratory phase, inspiration was delayed until the light stimulus was turned off (n = 6, Fig 4D).

The dependence of the stimulation effect on the respiratory phase was abolished when stronger stimuli (2.0 mW) where applied. The 2.0 mW laser pulses applied to the pre-BötC during the inspiratory phase reliably terminated the current inspiratory burst (n = 9, Fig 4G) and initiated rebound activation of inspiration leading to an advanced onset of the next inspiratory burst (n = 9, Fig 4E). Similarly, such stronger stimuli delivered during the expiratory phase caused rebound activation and advanced onset of the next inspiratory burst (n = 6, Fig 4F). Rebound activation, whenever it occurred, showed a consistent latency of 300 ms on average that was independent of the stimulation intensity (n = 6, Fig 4H).

### Effects of sustained photostimulation of VGAT-expressing inhibitory neurons within the pre-BötC

**Fig 5:**
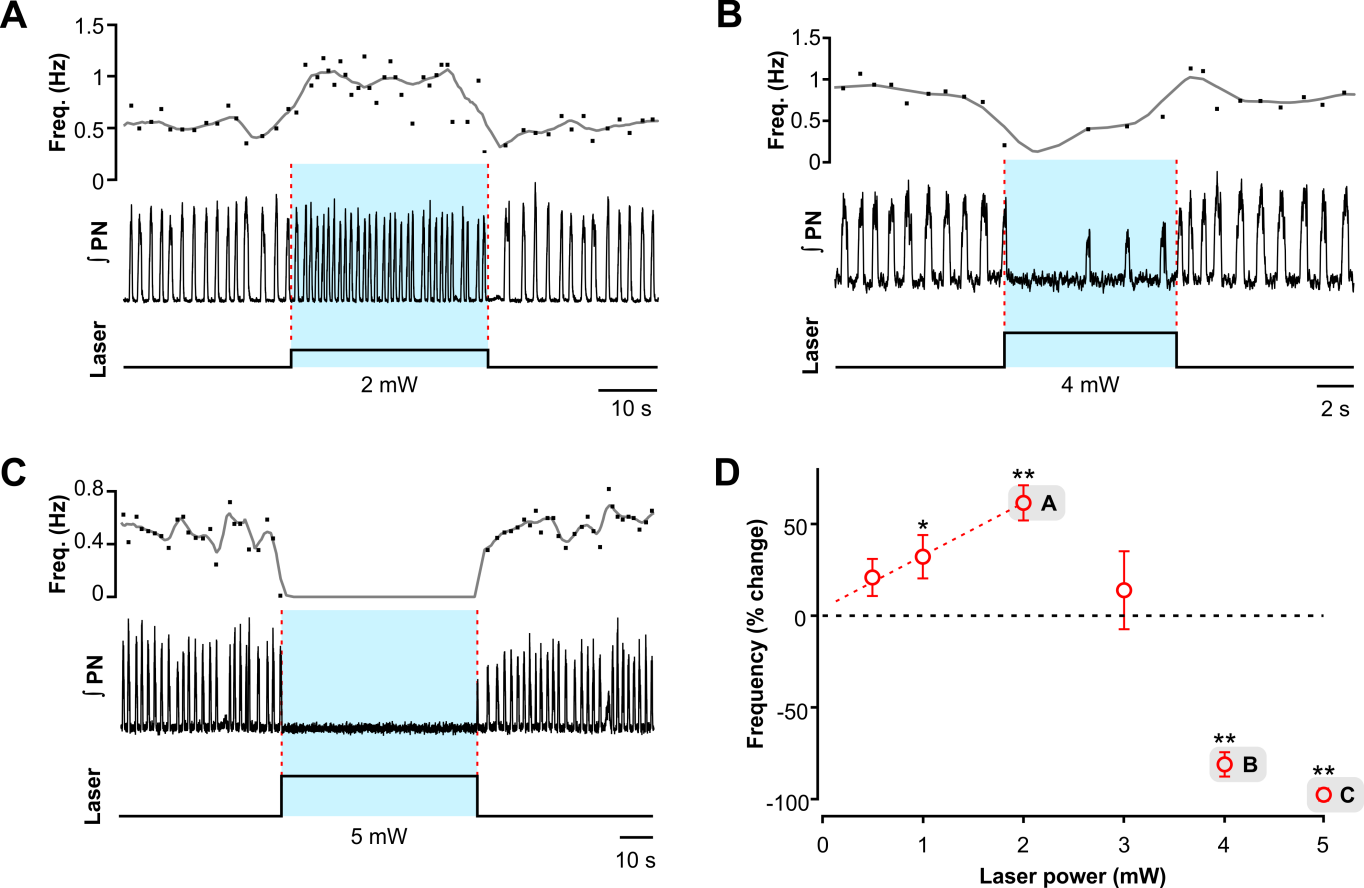
Effects of site-specific sustained photostimulation of inhibitory neurons within the pre-Bötzinger complex *in situ*. (**A-C**) Examples of the effects of sustained stimulation (473 nm, 20 Hz, 20 ms pulses) of low (2.0 mW laser power in A) and higher intensity (4.0 mW in B and 5.0 mW in C). Representative traces of integrated phrenic nerve recordings (∫PN) are shown in the middle; the upper traces show the instantaneous inspiratory frequency (dots) and its low pass filtered time course (time-based moving median in a 3 s window, solid black line, the lower traces indicate the duration and amplitude of the sustained laser stimulation epoch. (**D**) Summary data (n = 10, mean ± SEM, **p<0.01, *p<0.05) of bilateral pre-BötC photostimulation shows that relatively weak (0.5–2.0 mW) stimulation significantly increased respiratory frequency in a laser power-dependent manner, and stronger (4.0–5.0 mW) stimulation caused a decrease of inspiratory frequency with complete termination of inspiratory activity for the stimulus duration at the maximum laser power (5.0 mW).

To investigate how a sustained stimulation of pre-BötC inhibitory neurons affects the respiratory activity, we applied epochs (duration 10–60 s) of sustained laser illumination (20 Hz, 20 ms pulse trains, laser power of 0.5–5.0 mW) allowing a recovery period (> 4min) after each epoch. Bilateral laser illumination of pre-BötC regions caused rapid and reversible perturbations of the respiratory frequency (monitored by phrenic nerve (PN) or pre-BötC inspiratory activity recordings) as a function of laser power (0.5–5.0 mW range). Relatively weak sustained stimulation (0.5–2.0 mW) increased respiratory frequency (Fig 5A for a representative example of 2.0 mW stimulation). With further increase in the stimulation intensity, the frequency started to decrease (Fig 5D). The respiratory frequency was significantly reduced at 4.0 mW (Fig 5B) and the respiratory rhythm was abolished at a stimulation intensity of 5.0 mW (Fig 5C). The relationship between the respiratory frequency and stimulation intensity is illustrated in Fig 5D (n = 10, mean ± SEM, **p < 0.01, *p < 0.05) showing that bilateral activation of inhibitory neurons in the pre-BötC region affects the respiratory frequency in a photostimulation intensity-dependent manner. At relatively low stimulation intensity (laser power of 0.5–2.0 mW) frequency increased monotonically (linear regression fit: slope = 27.63 ± 7.39% per mW, r = 0.80), However, no significant increase of respiratory frequency was observed at 3.0 mW, and the stronger laser stimulation (4.0–5.0 mW) caused a significant decrease of frequency with termination of respiratory activity for the duration of the photostimulation at the maximum laser power (5.0 mW).

### Perturbations of the respiratory rhythm by short-duration photostimulation of VGAT-expressing neurons within the BötC

**Fig 6:**
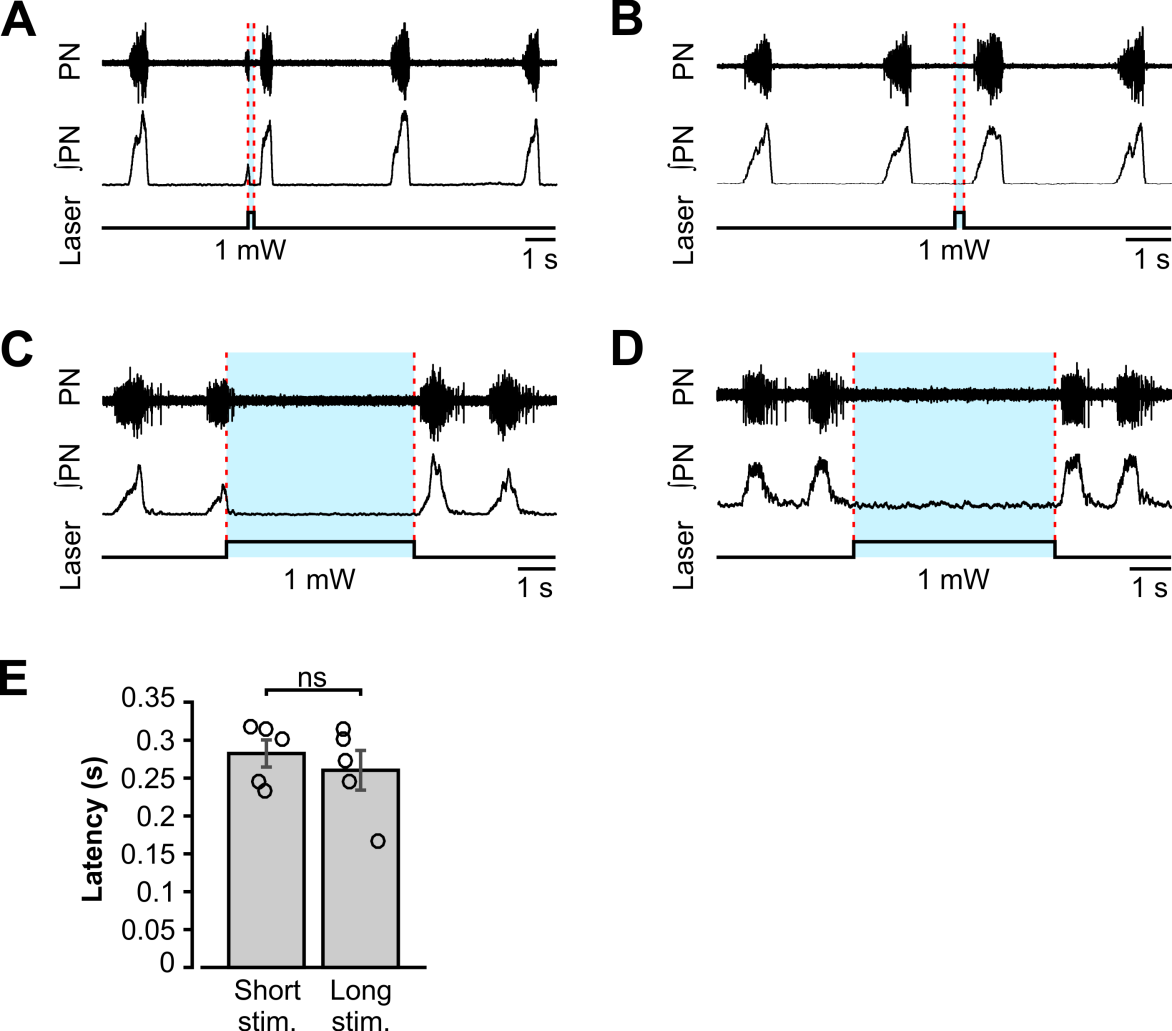
Perturbations of the inspiratory rhythm by bilateral photostimulation of VGAT-expressing inhibitory neurons within the Bötzinger complex *in situ*. (**A-D**) Representative traces of phrenic nerve (PN) inspiratory activity show the response to short light pulses activating ChR2 in BötC inhibitory neurons. The upper traces show raw PN recordings, the middle traces show integrated phrenic activity (∫PN), and the bottom traces show the laser stimulus application with the laser intensity given below the stimulus. The timing of the laser stimulation is indicated with blue shading and dashed red lines. (**A**) Short (200 ms) low-intensity (1.0 mW) photostimulation pulse applied during the inspiratory phase terminated inspiration and elicited rebound excitation of inspiratory activity after the end of stimulation. (**B**) The same stimulation during the expiratory phase also caused rebound excitation of the next inspiratory phase after the end of the stimulus. (**C** and **D**) Long (5s) duration single photostimulation epochs inhibited the respiratory rhythm for the duration of light application and elicited rebound excitation of inspiratory activity similar to the short stimuli in panels A and B. (**E**) Population data (n = 5, mean ± SEM) shows that the latency between the end of the light stimulus and the onset of the next inspiration was independent of the stimulus duration. Short stim. = 200 ms; Long stim. = 5 s.

To investigate the involvement of the BötC inhibitory neurons in the generation and control of the respiratory pattern, we applied bilateral site-specific photostimulation of VGAT expressing BötC neurons. In contrast to the intensity- and phase-dependent effects of pre-BötC stimulations, the effect of single light pulses applied to the BötC terminated inspiratory activity with photostimulation at low laser power (1.0 mW, n = 5, Fig 6), independent of the phase of application. The respiratory activity was terminated for the entire duration of the photostimulation epoch (Fig 6A–6D) and a release of light stimulation induced rebound activation of inspiration after a fixed latency of approximately 300 ms (Fig 6E), very similar to the rebound activation after stimuli applied to the pre-BötC.

### Effects of sustained photostimulation of VGAT-expressing inhibitory neurons within the BötC

**Fig 7:**
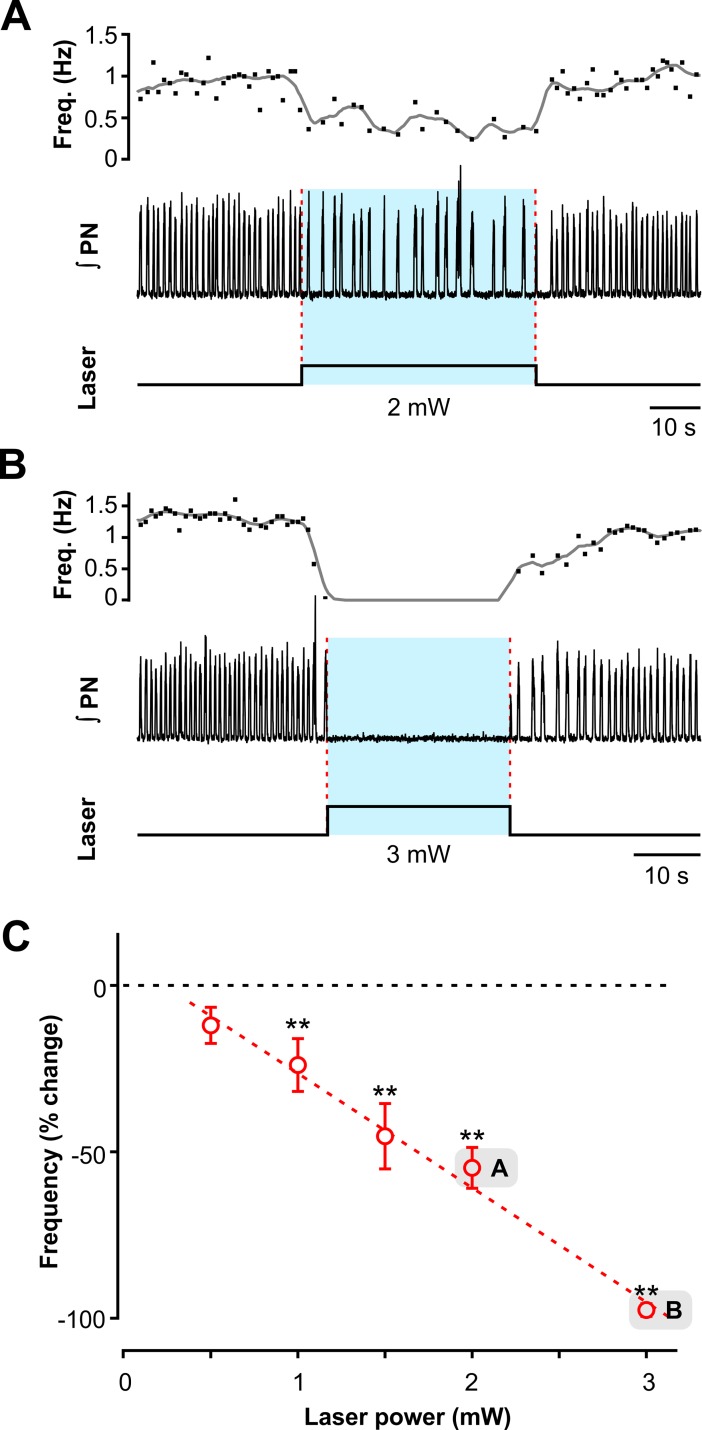
Effects of site-specific sustained photostimulation of inhibitory neurons within the Bötzinger complex *in situ*. (**A** and **B**) Examples of the effects of sustained stimulation (473 nm, 20 Hz, 20 ms pulses) of low (2.0 mW pulse amplitude in A) and higher intensity (3.0 mW in B). Representative traces of integrated phrenic nerve recordings (∫PN) are shown in the middle; the upper traces show the instantaneous respiratory frequency (dots) and its low pass filtered time course (time-based moving median in a 3 s window, solid black line), the lower traces indicate the duration and amplitude of the pulsed sustained laser stimulation. (**C**). Summary data (n = 12, mean ± SEM, ** p<0.01) show that bilateral BötC laser application significantly reduced respiratory frequency in a laser power-dependent manner with a complete cessation of the inspiratory rhythm at 3.0 mW.

Sustained optogenetic activation of BötC ChR2-expressing inhibitory neurons (20 Hz pulses, 0.5–3.0 mW, 30–60 sec duration) caused a rapid and reversible reduction of inspiratory frequency (Fig 7A for a representative example of the effect of 2.0 mW stimulation) with complete cessation of the rhythm at a maximum laser power of 3.0 mW (Fig 7B). Fig 7C (n = 12, mean ± SEM, ** p < 0.01) illustrates that bilateral sustained activation of BötC inhibitory neurons monotonically reduced inspiratory frequency in a laser power-dependent manner (0.5–3.0 mW, linear regression fit: slope = -35.36 ± 2.28% per mW, r = -0.89).

### Effects of sustained photostimulation in the BötC region on BötC post-I and aug-E neuron activity and inspiratory neuron activity in the pre-BötC *in situ*

**Fig 8:**
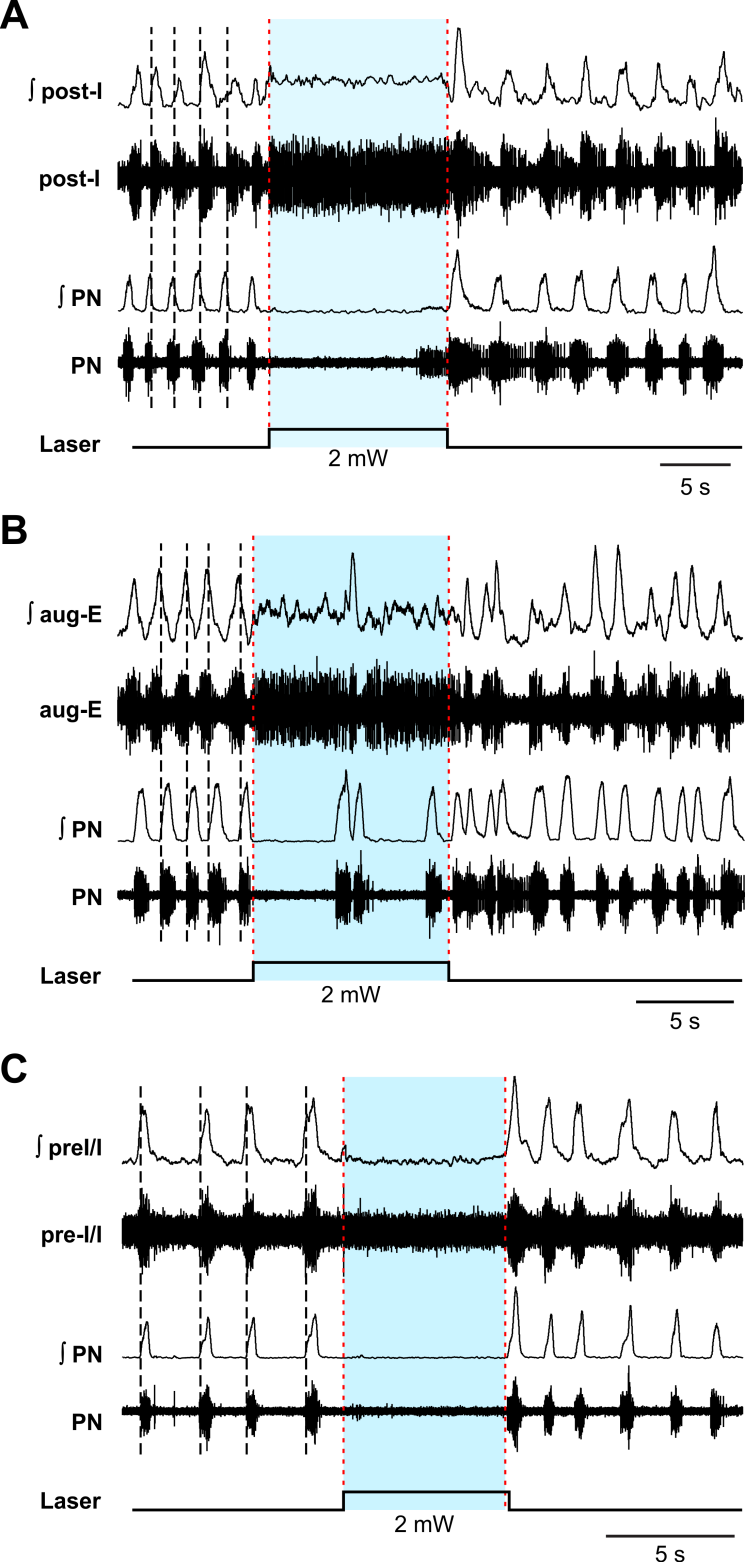
Photostimulation in the BötC region causes excitation of BötC post-I and aug-E neuron activity and inhibition of inspiratory neuron activity in the pre-BötC *in situ*. **(A)** Extracellular recordings from post-I type expiratory neurons in the BötC showing example of tonic excitation of neuron activity during laser application to the BötC (2 mW) along with complete inhibition of inspiratory phrenic nerve activity (PN) in the arterially-perfused *in situ* brainstem-spinal cord preparation from adult VGAT-ChR2 transgenic mice. **(B)** Extracellular recordings from aug-E type expiratory neurons in the BötC also showing excitation of neuron activity during laser illumination (2 mW) of the BötC region, which reduced the frequency of inspiratory activity in this example. (**C**) Extracellular recordings from inspiratory (pre-I/I) neuron activity in the pre-BötC illustrating inhibition of PN inspiratory activity by 2 mW laser illumination in the BötC region. Black dashed lines indicate the end of inspiratory phase activity of PN in A, but indicate the onset of inspiratory phase activity of PN in B and C. The lower traces indicate the duration and amplitude of the laser stimulation.

To investigate the effects of photostimulation (20 Hz pulses, 0.5–3.0 mW) in the BötC region on the activity of BötC and pre-BötC respiratory neurons, we recorded extracellular population activity from BötC post-I or aug-E type of expiratory neurons as well as pre-BötC pre-I/I type inspiratory neurons in perfused brainstem-spinal cord *in situ* preparations from adult VGAT-ChR2 mouse lines. Extracellular recordings from post-I expiratory neurons in the BötC (n = 3, Fig 8A) showed a rapid and reversible augmentation, typically tonic excitation of neuron activity during laser application at ≥ 2 mW laser power to the BötC, which also caused reduction of inspiratory frequency and inhibition of inspiratory PN activity. In aug-E type of expiratory neurons in the BötC (n = 4, Fig 8B), photostimulation to the BötC similarly caused augmentation of neuron activity along with reduction of inspiratory frequency and inhibition of inspiratory PN activity. These results indicate that laser illumination (0.5–3.0 mW) in the BötC region induced excitatory effects on expiratory VGAT-positive inhibitory neurons in the BötC. On the other hand, extracellular recordings from inspiratory (pre-I/I) neuron activity in the pre-BötC (n = 6, Fig 8C) showed inhibition, typically complete silencing, of neuron activity during laser application to the BötC, which also caused a reduction of inspiratory frequency and inhibition of inspiratory PN activity. These results suggest functional inhibitory connections from BötC to pre-BötC circuits, consistent with the core respiratory network configuration proposed in our computational model (see below).

### A revised computational model of neural interactions within the pre-BötC/BötC core network reproduces the effects of site-specific selective activation of inhibitory neurons in the pre-BötC and BötC

**Fig 9:**
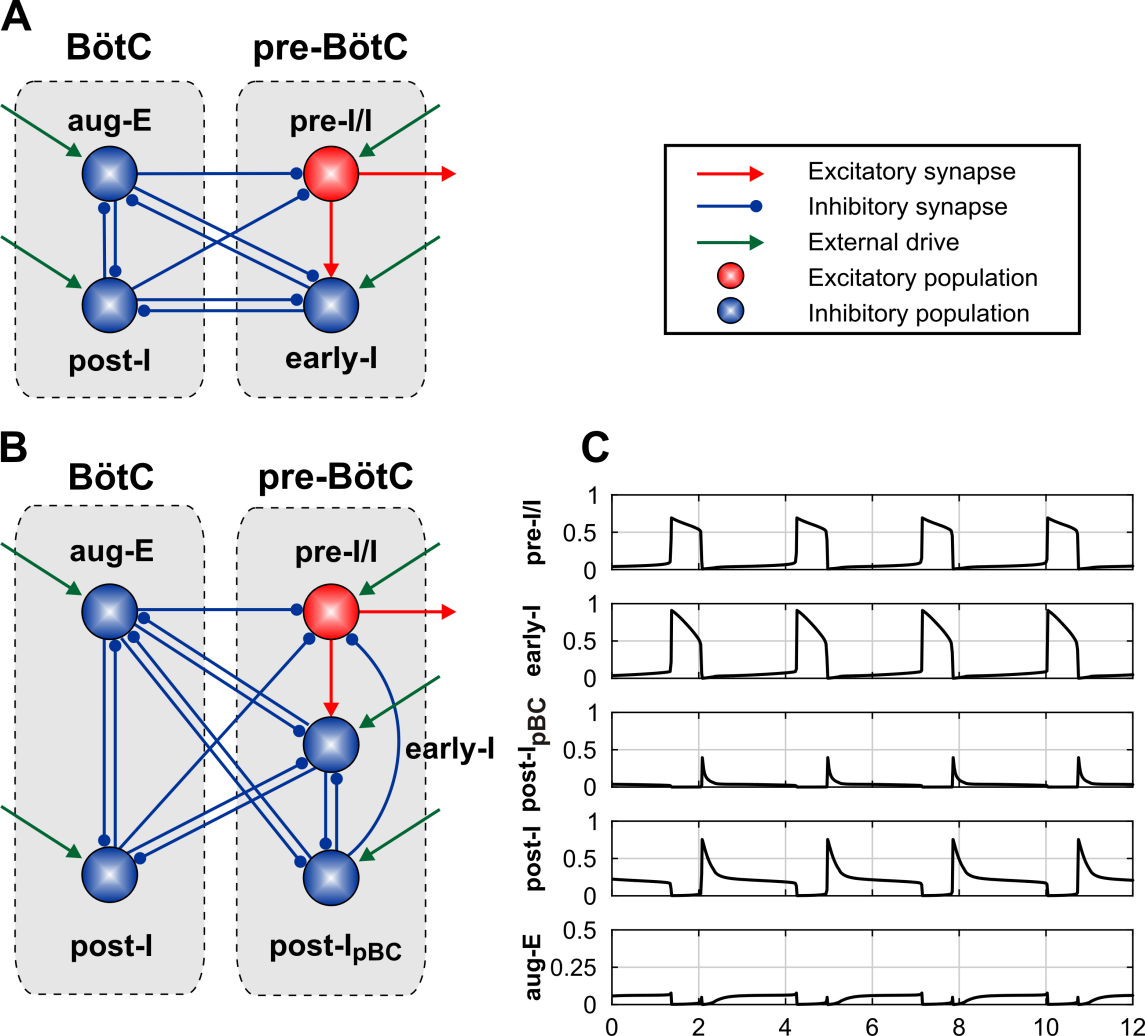
Schematic of neural interactions within the pre-BötC-BötC core network and model performance. (**A**) Schematic of the pre-BötC-BötC respiratory network from the model of Rubin et al. [10]. (**B**) Model schematic of the proposed extended model that includes the novel post-I_pBC_ population within the pre-BötC. (**C**) Model performance under normal conditions (without stimulations). The traces represent output activity of each population in the network.

Previous models of the brainstem respiratory circuits [3,4,6–8,10] proposed neural interactions within and between the pre-BötC and BötC as shown in Fig 9A. In these models, the pre-BötC contained only populations of inspiratory neurons: the excitatory pre-inspiratory/inspiratory (pre-I/I) and the inhibitory early-inspiratory (early-I) populations. The BötC contained the inhibitory post-inspiratory (post-I) and augmenting expiratory (aug-E) populations. Hence, post-I neurons were suggested to be only present in the BötC but not in the pre-BötC. However, our analysis has shown that this architecture of the pre-BötC/BötC core network is unable to reproduce and explain the above experimental data on phase- and stimulation intensity-dependent effects of site-specific activation of inhibitory neurons within the pre-BötC. This conclusion was based on the following. First, optogenetic stimulation of the pre-BötC inhibitory neurons revealed a rebound activation of inspiration after the end of a sufficiently strong photostimulus (Fig 4B–4F). The excitatory pre-I/I population could, in principle, generate a rebound activation, because of the persistent sodium-dependent mechanism. However, the pre-BötC compartment in previous models contained only the early-I inhibitory population (Fig 9A) which was active during inspiration only, did not inhibit pre-I/I neurons, and thus could not cause rebound activation of pre-I/I neurons. Second, we showed that the effect of low-intensity photostimulation of inhibitory pre-BötC neurons was suppressed during inspiration (Fig 4A), but this suppression could be overcome by a stronger light activation (Fig 4E). Together these observations suggest that the neurons responsible for termination of inspiration could be inhibited during the inspiratory phase unless the intensity of laser stimulation overcame this inhibition. Finally, additional activation of early-I inhibitory neurons in the previous models was known to increase respiratory frequency (e.g. [10]) and thus could not account for the biphasic shape of the frequency response to sustained light activation of inhibitory pre-BötC neurons (Fig 5A).

Further analysis of the previous models in the context of our new data led us to the suggestion that the above effects can be reproduced and explained by having post-inspiratory neurons located in both the BötC and the pre-BötC. Indeed, neurons with post-inspiratory firing characteristics have been previously found in the pre-BötC [9,26,27]. Therefore, we extended the previous models by incorporating an additional population of post-inspiratory inhibitory neurons in the pre-BötC and called it post-I_pBC_, (Fig 9B). Except for its location, the proposed inhibitory post-I_pBC_ population was similar to the post-I population in the BötC: it had adapting intrinsic properties and inhibited all other populations in the network except for its partner post-I population in the BötC. Like the BötC's post-I population, the post-I_pBC_ population had mutual inhibitory interactions with the early-I population of the pre-BötC and the aug-E population of the BötC (Fig 9B). To match model behavior to the experimental data, we assumed that the post-I_pBC_ strongly inhibits the pre-I/I and other respiratory populations leading to rebound excitation of inspiration after ending the post-I_pBC_ photostimulation (Fig 4B–4D). The dependence of network behavior on the intensity of the applied photostimulation led us to the suggestion that the observed different (and sometimes opposite) consequences of weak vs. strong stimulations may result from mutual inhibitory interactions between the post-I_pBC_ and early-I populations of the pre-BötC, so that the early-I inhibits post-I_pBC_ at lower stimulation intensities, whereas post-I_pBC_ overcomes this inhibition at higher stimulation intensities. Activation of ChR2 by photostimulation was implemented in the model as opening an additional excitatory "ChR2" channel in the populations of inhibitory neurons within the compartment (pre-BötC or BötC) stimulated by light. The light sensitivity of each inhibitory population was defined by the conductance of this channel (*g*_*ChR*_, which was the same in each inhibitory population, see Methods). The intensity of light stimulation was characterized by the parameter “*stim*_*ChR*_“. Incorporating the *g*_*ChR*_, conductance in all inhibitory neurons allowed the above mutual inhibitory interactions between the post-I_pBC_ and early-I populations. Specifically, at relatively low stimulation intensity during inspiration, the early-I population (receiving excitation from the pre-I/I population) kept the post-I_pBC_ population inhibited, and thus did not allowing the applied stimulation to terminate inspiration. In this case, the early-I population became highly active and close to saturation of its activity. Consequently, an additional increase of stimulation intensity affected early-I activity less than that of the post-I_pBC_ population. These different degrees of additional activation by photostimulation allowed the post-I_pBC_ population to overcome early-I inhibition at higher stimulation intensity and to inhibit both early-I and pre-I/I populations, hence terminating inspiration.

Network operation under normal conditions is shown in Fig 9C. During expiration, the BötC post-I population demonstrates adapting (decrementing) activity (see Methods). The corresponding decline in inhibition from this population shapes the augmenting profile of aug-E activity. The reduction in post-I inhibition also produces slow depolarization of pre-I/I and early-I neurons in the pre-BötC. In addition, the pre-I/I population is depolarizing because of the slow deinactivation of the persistent (slowly inactivating) sodium current (*I*_*NaP*_, see Methods). At some moment during expiration, the pre-I/I population rapidly activates, providing excitation of early-I. The latter inhibits post-I, aug-E and post-I_pBC_ populations, completing the switch from expiration to inspiration. During inspiration, the early-I population of the pre-BötC demonstrates adapting (decrementing) activity. The decline in inhibition from this population produces a slow depolarization of the post-I, aug-E and post-I_pBC_ populations. At some moment, the post-I populations of BötC rapidly activates and inhibits both inspiratory neurons (pre-I and early-I) and the aug-E neuron (initially), producing the switch from inspiration to expiration and the release of the fast adapting post-I_pBC_ population in the pre-BötC.

### Modeling the effects of selective activation of inhibitory neurons within the pre-BötC

**Fig 10:**
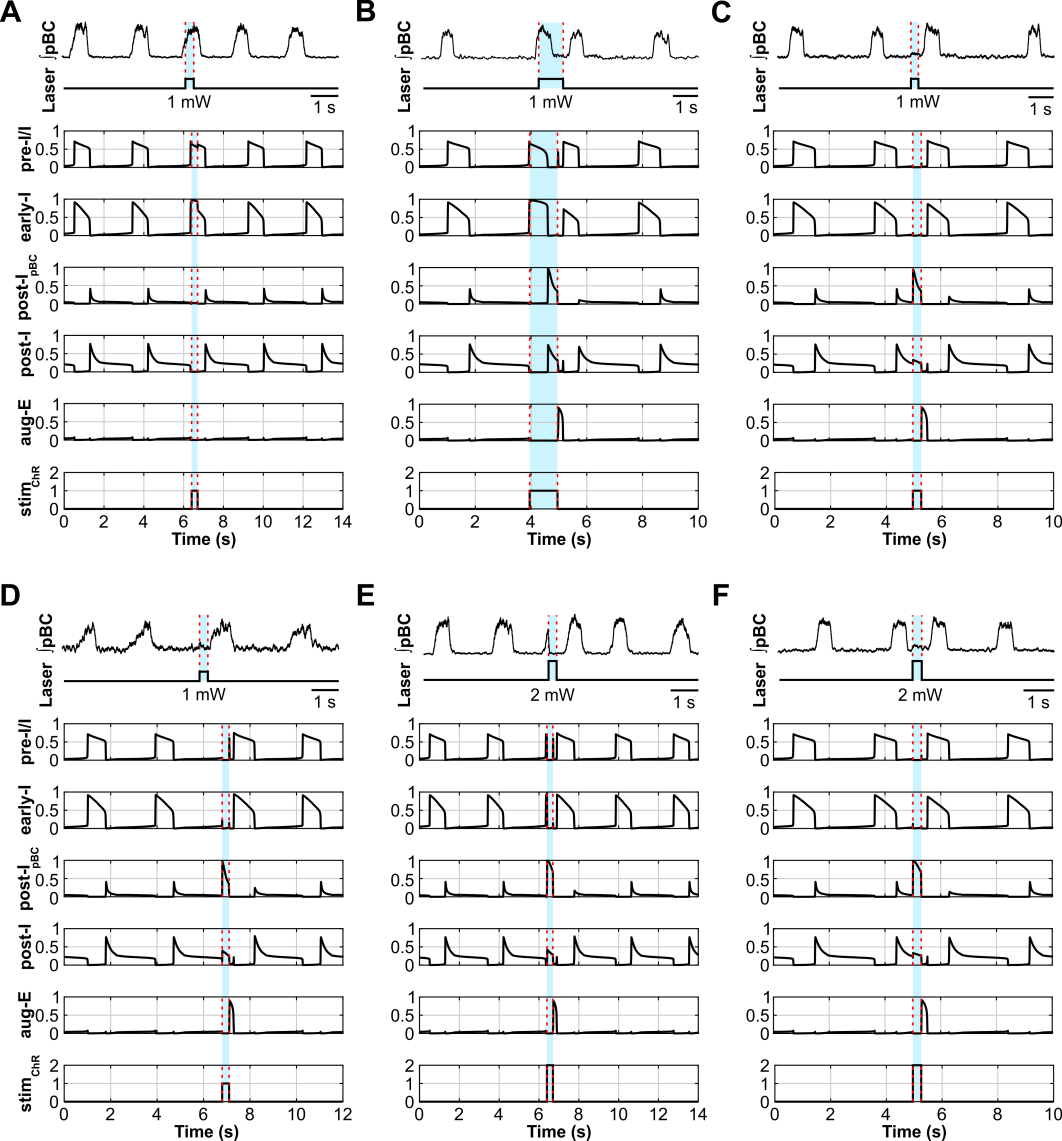
Model performance in comparison to experimental data: Responses to short stimuli activating inhibitory neurons within the pre-BötC. (**A-F**) The upper two traces illustrate the effects of photostimulations in our experiments from the examples shown in Fig 4. The lower five traces show simulated activity of all neuron populations in the model. The applied stimulations are shown at the bottom. The timing of the laser stimulus is indicated with blue shading and dashed red lines. The model reproduces the behavior of the neurobiological system under all experimental situations shown above. The description of the different perturbations is the same as in the corresponding panels of Fig 4.

The model reproduced our experimental findings on the effects of selective optogenetic activation of inhibitory neurons in the pre-BötC (Fig 10). Similar to our experimental data, activating both inhibitory populations in the pre-BötC (early-I and post-I_pBC_) in the model by the simulated weak pulses (300 ms, *stim*_*ChR*_ = 1) did not terminate inspiration and did not affect the respiratory rhythm (Fig 10A). The weak pulses induced rebound activation advancing the next inspiratory activity if the stimulus outlasted the inspiratory phase (1 s, *stim*_*ChR*_ = 1, Fig 10B) or was applied during the expiratory phase (300 ms, *stim*_*ChR*_ = 1, Fig 10C). Also, inspiratory phase activity was delayed until the light stimulus was turned off, when the stimulus occurred very late during the expiratory phase and lasted into the next inspiratory phase (300 ms, *stim*_*ChR*_ = 1, Fig 10D).

Similar to our experimental data, stronger stimuli (300 ms, *stim*_*ChR*_ = 2) applied during the inspiratory phase terminated inspiratory activity (Fig 10E). Strong stimuli during either the inspiratory or expiratory phase initiated rebound activation of inspiration and advanced the next inspiratory activity phase (Fig 10E and 10F). As seen in Fig 10B–10F termination of the stimulus first produced a release of the aug-E population of the BötC, whose activity created a fixed latency for release of activity of the pre-BötC pre-I/I population that defines the inspiratory phase.

**Fig 11:**
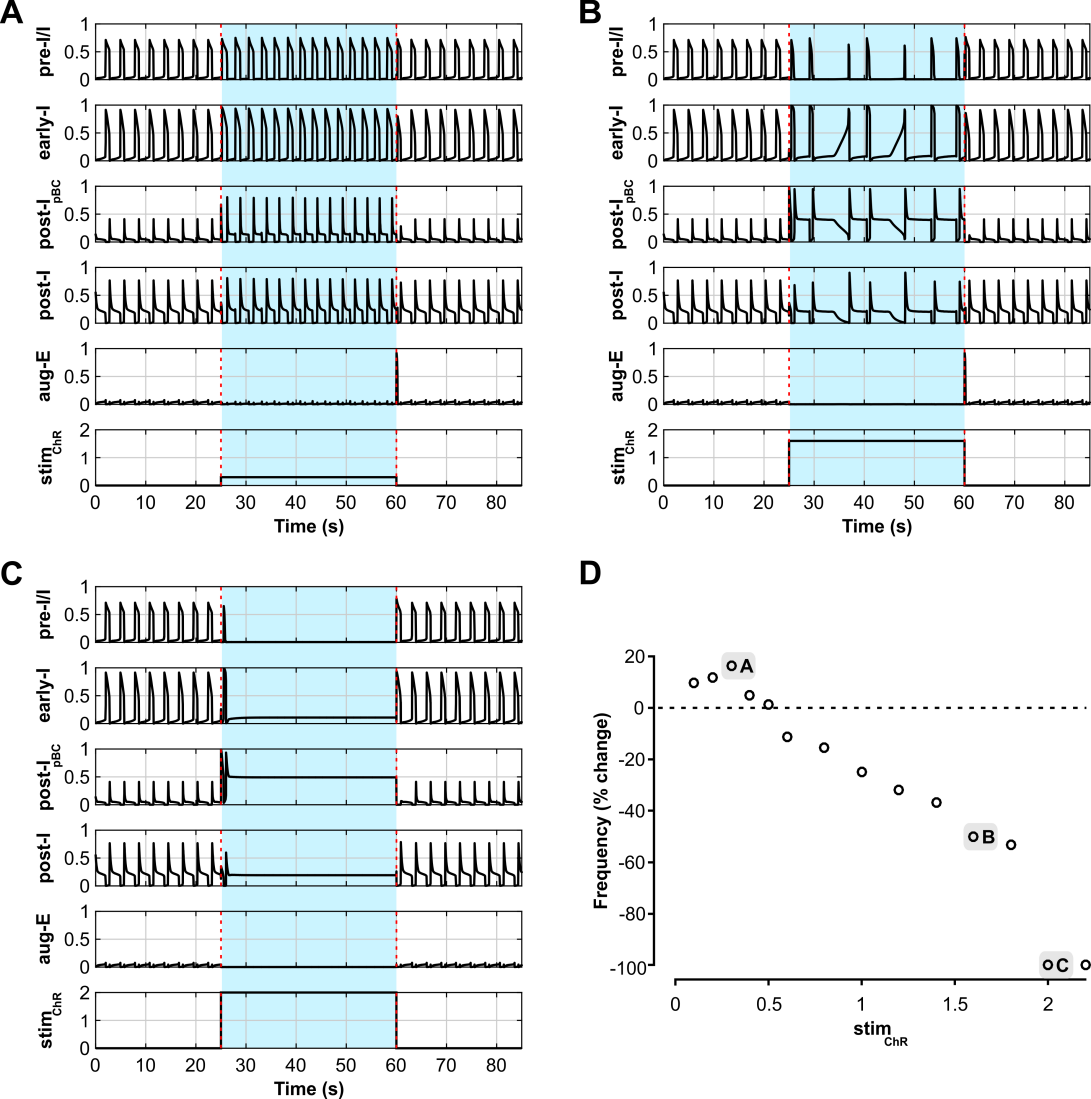
Model performance compared to experimental data: responses to sustained stimulations of inhibitory neurons within the pre-BötC. (**A-C**) Model responses to sustained stimulation of inhibitory neurons within the pre-BötC with low-intensity (A; *stim*_*ChR*_ = 0.3) and higher intensity (B, *stim*_*ChR*_ = 1.6, and C, *stim*_*ChR*_ = 2) stimulation. The timing of the stimulus is indicated with blue shading and dashed red lines. Output activity of all neuron populations in the model are shown; the oscillation frequency increases in A, decreases in B, and oscillations are completely suppressed in C, similarly to our experimental studies (Fig 5) (**D**) Biphasic change in oscillation frequency in the model depending on the stimulation intensity.

The model also reproduced the biphasic frequency response of a sustained activation of inhibitory neurons within the pre-BötC. Sustained activation of early-I and post-I_pBC_ inhibitory populations increased the frequency at low stimulation intensities (*stim*_*ChR*_ ≤ 1, Fig 11A), whereas further increase of stimulus intensity reduced the frequency (*stim*_*ChR*_ > 1, Fig 11B) and finally terminated respiratory oscillations (*stim*_*ChR*_ > 1.8, Fig 11C). The intensity range of the sustained stimulation in the model was reduced by a factor of two (Fig 11C), which is consistent with the level of inactivation under continuous light stimulation of ChR2 channels in the biological system [49,50].

### Modeling the effects of selective activation of inhibitory neurons within the BötC

**Fig 12:**
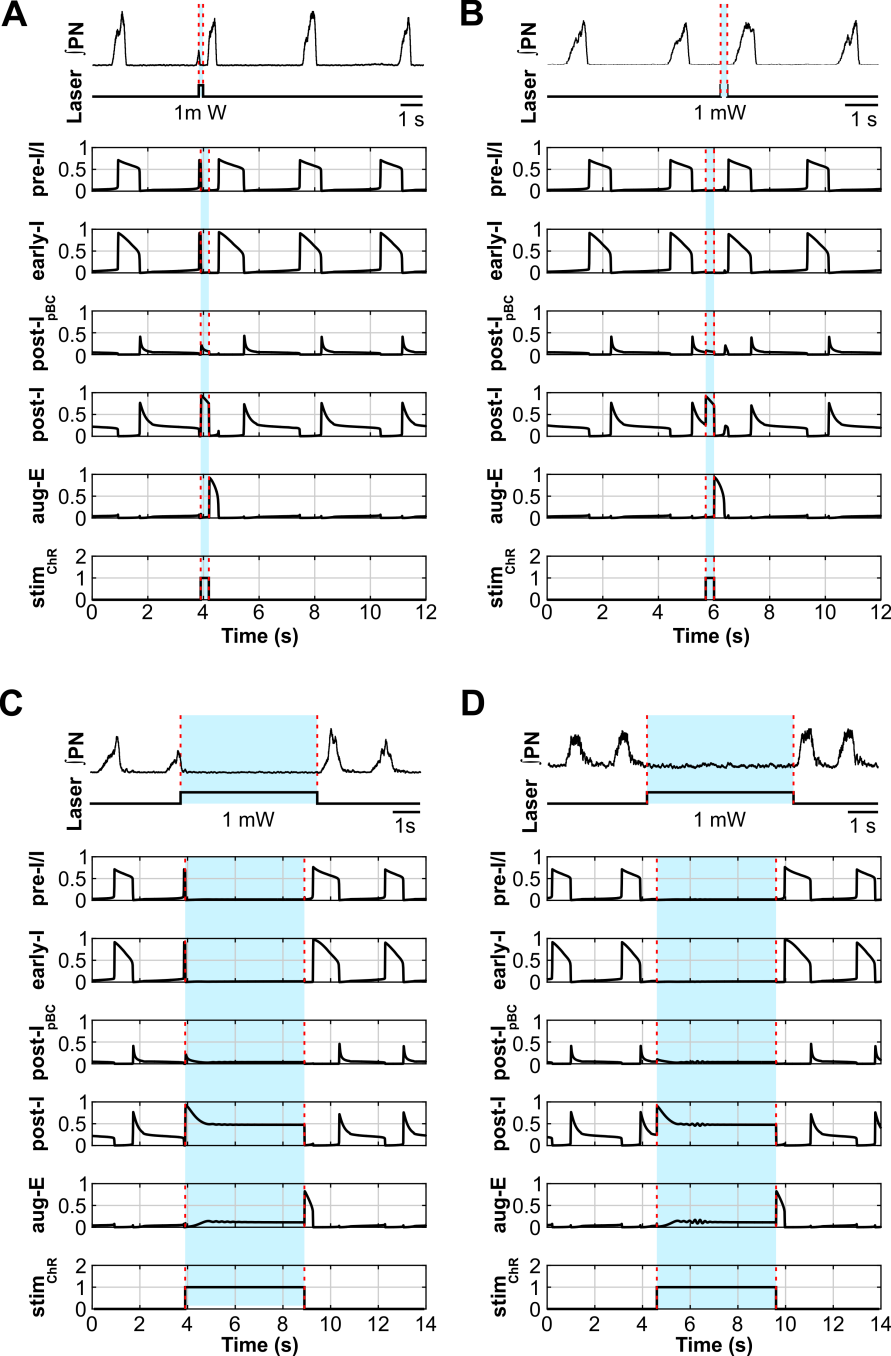
Model performance vs. experimental data for responses to short stimuli activating inhibitory neurons within the BötC. (**A-D**) The upper two traces illustrate the effects of photostimulations in our experiments from the examples shown in Fig 6. The lower five traces show the simulated activity of all neuron populations in the model. The applied stimulations are shown at the bottom. The timing of the laser stimulus is indicated with blue shading and dashed red lines. The model reproduces the behavior of the biological system under all experimental situations shown above. Description of the different perturbations is the same as in the corresponding panels of Fig 6.

Similar to our experimental studies, sustained activation of inhibitory neurons within the BötC (aug-E and post-I) by short (300 ms) and long (5 s) single pulses inhibited the respiratory rhythm for the duration of each pulse (Fig 12) and the termination of each stimulus first produced a transient release of activity of the BötC aug-E population which delayed the onset of the next inspiratory phase with a latency (Fig 12A–12D) consistent with our experimental studies.

**Fig 13:**
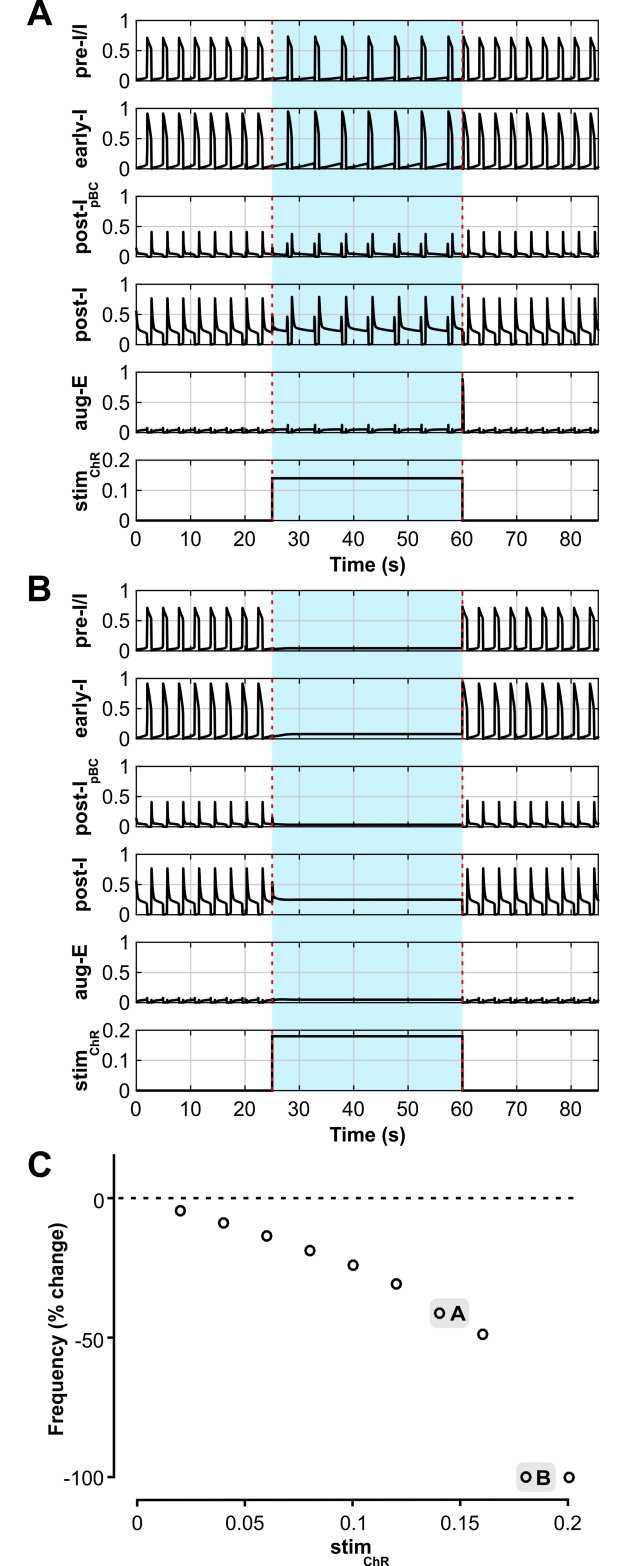
Model performance vs. experimental data for responses to sustained stimulations of inhibitory neurons within the BötC. (**A** and **B**) Model responses to sustained stimulation of inhibitory neurons within the pre-BötC with low-intensity (A; *stim*_*ChR*_ = 0.14) and higher intensity (B, *stim*_*ChR*_ = 0.18) stimulation. The timing of the stimulus is indicated with blue shading and dashed red lines. Output activity of all neuron populations in the model are shown. The oscillation frequency decreases maximally in A, and is fully suppressed in B, similar to our experimental results (Fig 7) (**C**). Increases of stimulation intensity reduced oscillation frequency and terminated inspiratory activity at the highest intensities examined, directionally similar to the experimental results shown in Fig 7C.

Also, similar to the experimental results, a sustained activation of inhibitory populations within the BötC decreased the respiratory frequency with low stimulation intensities (*stim*_*ChR*_ < 0.18, Fig 13A and 13C) and suppressed all activity with higher intensities (*stim*_*ChR*_ ≥ 0.18, Fig 13B and 13C), albeit at a lower intensity range when compared to the short pulses and to the pre-BötC stimulation.

### Modeling the effects of site-specific optogenetic stimulation of all neurons within the pre-BötC or BötC

**Fig 14:**
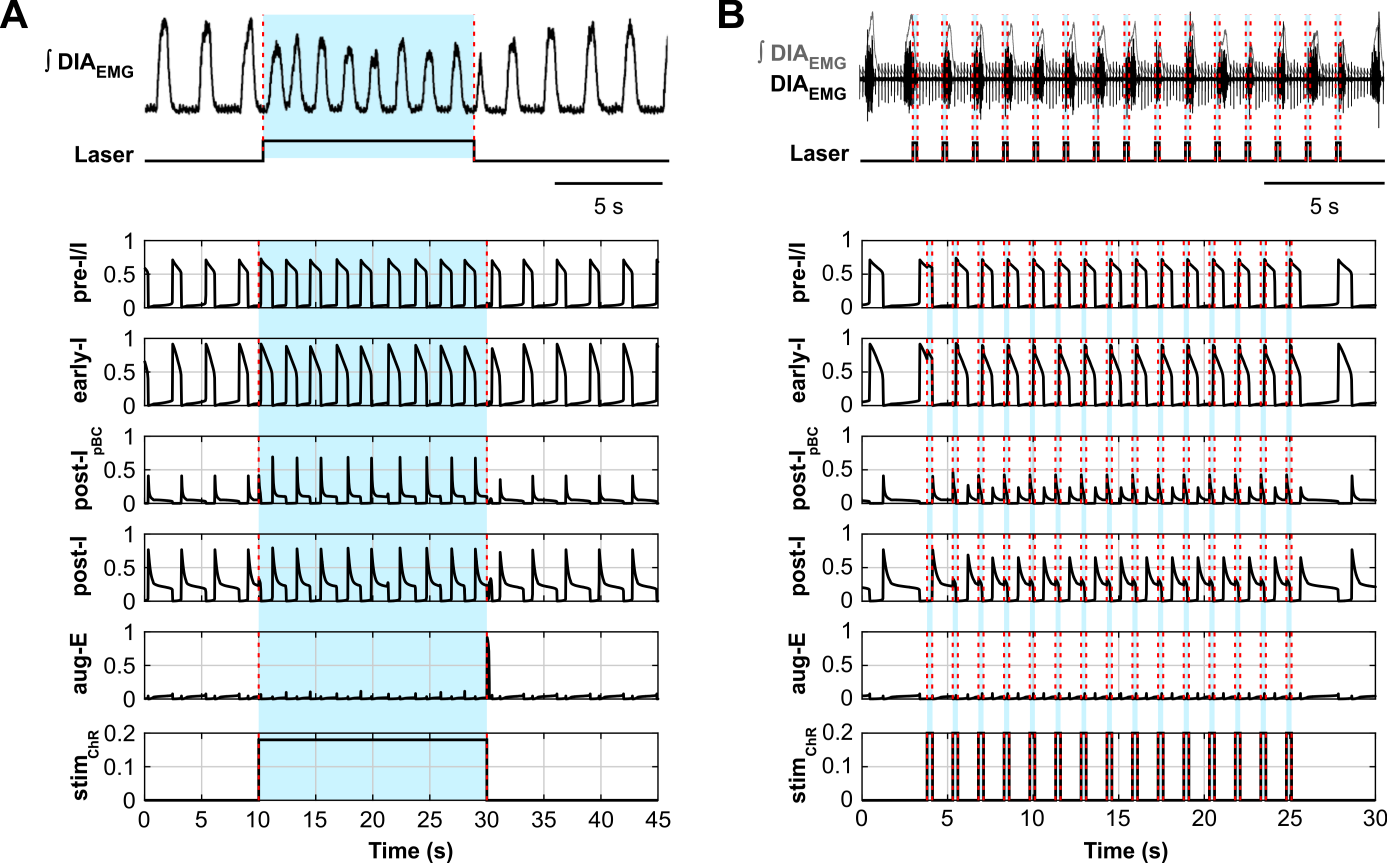
Model-data comparisons for responses to stimulations of inhibitory and excitatory pre-BötC populations. (**A**) Integrated recording of diaphragmatic EMG (ʃDIA_EMG_) activity and laser activation illustrating that continuous ChR2 photostimulation of excitatory and inhibitory pre-BötC together induces an increase in respiratory frequency (data from the study of Alsahafi et al. [47], Fig 2B, used with authors’ permission). The lower five traces show the activity of all neuron populations in the model. The applied photostimulations are shown at the bottom (*stim*_*ChR*_ = 0.18). The timing of the laser stimulus is indicated with blue shading and dashed red lines. Stimulating all populations in the model elicited a frequency increase similar to the experimental data. (**B**) Recording of ʃDIA_EMG_ and laser frequency during 1 Hz photostimulation showing entrainment of the respiratory rhythm to the periodic stimulation (data from the study of Alsahafi et al. [47], Fig 6B, used with authors’ permission). The lower five traces show the activity of all neuron populations in the model. The applied stimulations are shown at the bottom (*stim*_*ChR*_ = 0.2), and the timing of the laser stimulus is indicated with blue shading and dashed red lines. Stimulating all pre-BötC populations in the model can entrain the network activity at the experimental photostimulation frequency of 0.67Hz.

The computational model presented here reproduced our experimental findings on the effects of sustained and phase-dependent short-duration activation of inhibitory neurons in the pre-BötC. To validate the model performance further, we simulated the simultaneous optogenetic stimulation of all, inhibitory and excitatory, neurons within the pre-BötC and compared the modeling results with findings in a recently study of Alsahafi et al. [47]. They unilaterally activated pre-BötC or BötC neurons using an adeno-associated virus expressing ChR2 under the synapsin promoter to selectively photoactivate all (excitatory and inhibitory) neurons in the pre-BötC or the BötC [47]. Specifically, Alsahafi et al. [47] showed that the sustained stimulation of pre-BötC increased respiratory frequency (Fig 14A, top diagram) and that the network could be entrained by repetitive application of short light pulses (Fig 14B, top diagram).

To test the model against the experimental data published by Alsahafi et al. [47] we set the light sensitivity of the excitatory pre-I/I population equal to that of post-I_pBC_ and early-I populations (see Methods). Similar to the experimental results of Alsahafi et al. [47], sustained stimulation of all populations in the pre-BötC increased respiratory frequency in our model at *stim*_*ChR*_ = 0.18 (Fig 14A, bottom diagram). In the model, entrainment of the rhythm was possible down to a respiratory cycle period of 1.5 s with 300 ms pulses of *stim*_*ChR*_ = 0.2 to all populations of the model pre-BötC (Fig 14B, bottom diagram).

**Fig 15:**
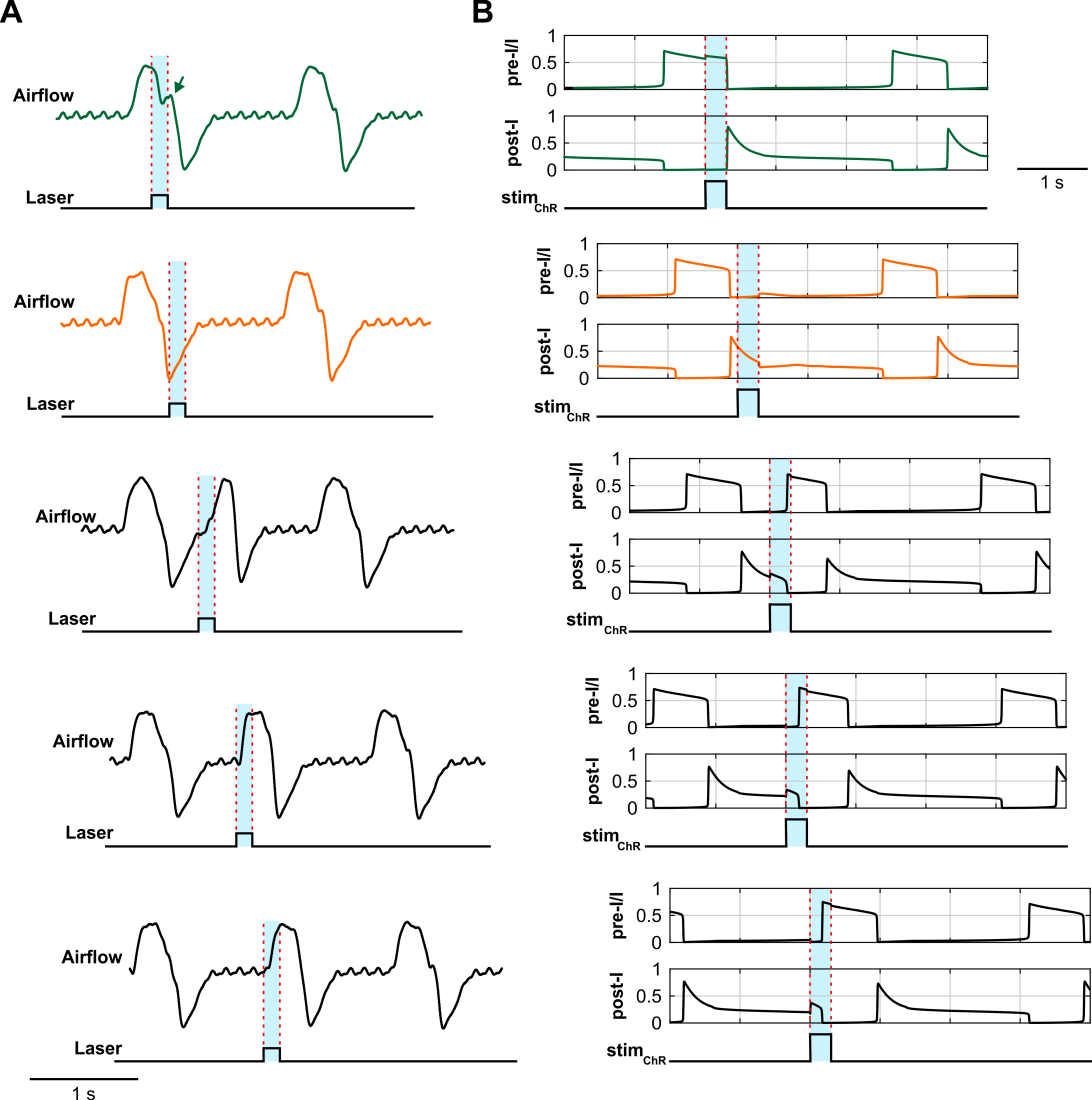
Data-model comparisons of respiratory phase dependent photostimulation of pre-BötC neurons. (**A**) Five respiratory airflow traces illustrating the response to stimulation of pre-BötC neurons at different respiratory phases. The orange trace indicates a lack of response to photostimulation when it occurs during the post-inspiratory phase (refractory period). The green trace shows a slight prolongation of inspiration (green arrow) when the stimulus is delivered during the inspiratory phase and the black traces show initiation of inspiration during the stimulus after a short latency (based on data from the study of Alsahafi et al. [47], Fig 7B, used with authors’ permission). (**B**) The model output traces of pre-I/I and post-I activities show a similar behavior to the experimental data when all pre-BötC populations are stimulated with a 300 ms simulated light pulse. The applied stimulations are shown at the bottom (*stim*_*ChR*_ = 0.2). The timing of the laser stimulus is indicated with blue shading and dashed red lines.

Alsahafi et al. [47] also demonstrated that optogenetic stimulation of pre-BötC neurons with short stimuli could induce a reliable reset of inspiratory activity but found a post-inspiratory period during which the rhythm could not be reset (Fig 15A). The reset of inspiration in their experiments occurred with a short delay (about 100 ms) after the stimulus onset. The post-inspiratory period during which optogenetic stimulation of the pre-BötC was unable to initiate the next inspiratory event was about 200 ms (Fig 15A). In our model, stimulating all neuron populations within the pre-BötC also initiated a reset of the inspiratory burst (with a slightly longer latency of ~200 ms) when stimulated at *stim*_*ChR*_ = 0.2 (Fig 15B). The post-I period insensitive for inspiratory resetting was about 300 ms and was mainly defined by the activity of the post-I population of the BötC and the time constant of its adaptation.

Our experimental and modeling results were also consistent with other data reported by Alsahafi et al. [47]. In some experiments, they used optogenetic excitation of BötC neurons which resulted in a decrease in respiratory frequency or complete suppression of inspiratory activity during the applied stimuli (Fig 4B and 4C in their paper). This corresponds to our experimental (Fig 7) and modeling (Fig 11) results. Thus, our model reproduces multiple results of experimental perturbations that employed selective optogenetic activation of either inhibitory neurons within the pre-BötC and BötC or of all neuron types in each of these compartments.

## Discussion

The goal of our study was to probe the circuit organization and neuron interactions within core circuits of the brainstem respiratory CPG network, particularly within the pre-BötC and BötC, and further define their roles in respiratory rhythm and pattern generation. Since inhibitory circuit interactions are critically involved, we used mice expressing Channelrhodopsin 2 (ChR2) under the VGAT promoter and performed optogenetic activation of inhibitory neurons (both glycinergic and GABAergic) by light stimuli applied separately to the pre-BötC and BötC compartments. The perturbations of respiratory rhythm and pattern produced by these photostimulations depended on the stimulus intensities and phases of their application in the respiratory cycle. Accordingly, we have analyzed these features to gain new insights into possible circuit organization in these compartments. Based on this analysis, an updated connectome of the pre-BötC and BötC has been proposed from computational modeling and validated by comparing the model performance to the experimental data from our and other laboratories.

### Validation of Cre-driver and Cre-driven optogenetic mouse lines, including neuron- and site-specificity of optical activation of inhibitory neurons within pre-BötC and BötC regions

We established a VGAT-tdTomato-ChR2-EYFP transgenic mouse line with Cre-driven ChR2 expression selectively in VGAT-positive neurons for temporally controlled, specific optogenetic manipulation of pre-BötC and BötC inhibitory neuron population activity. To validate this triple Tg line, we (1) verified the Cre-driver line (VGAT-Cre) used to derive this strain by tdTomato reporter expression, (2) performed immunocytochemical analyses of antibody co-labeling for glycine/GABA establishing that the majority of these tdTomato-labeled neurons were labeled by glycine or GABA antibodies, and subsequently (3) confirmed ChR2-EYFP fusion protein expression in the majority of VGAT-tdTomato labeled neurons in the pre-BötC and BötC regions as illustrated in Results (Figs 1 and 2).

An important consideration for interpreting our experimental results is whether our transgenic approach to drive expression of ChR2 is efficient for site-specific photostimulation of inhibitory neuron depolarization, which is a basic assumption of our modeling studies. In the mouse line used, the VGAT Cre-driven expression of ChR2 occurs not only in neuronal soma but also in processes/terminals of inhibitory neurons. This could confound the interpretation of our experimental results, since photostimulation of inhibitory synaptic processes on targeted inhibitory neurons could inhibit postsynaptic activity rather than stimulate somatic spiking of these neurons. To address this problem and validate our approach, we recorded neuronal responses to photostimulation by somatic whole-cell recording *in vitro*, and we also performed extracellular recordings of neuronal activity in more intact *in situ* brainstem-spinal cord preparations. The responses to photostimulation were always depolarization of rhythmic inspiratory VGAT-expressing neurons in the pre-BötC *in vitro*, as well as augmented spiking of rhythmic inhibitory neurons recorded extracellularly. For example, expiratory neurons in the BötC always extended their spiking when the optical cannulae are positioned directly over this region *in situ*. If activation of ChR2 in terminals from inhibitory neurons located elsewhere, or within local circuits, that synapse on the inhibitory neurons in the BötC or pre-BötC regions was the predominant response to photostimulation, then we would expect to observe neuronal hyperpolarization or inhibition of neuronal spiking activity, which is clearly not the case. If photoactivation of inhibitory terminals is involved to some extent, it is certainly possible that the light-induced depolarization/spiking that we consistently observed may be somewhat attenuated, relative to the case where only ChR2 channels in neuronal somal membranes are activated. In general, however, our experimental tests indicate that with the VGAT-Cre transgenic approach, we achieve a net photo-induced depolarization/spiking of inhibitory neurons, which is consistent with the assumptions of the modeling.

It should be noted that increasing the intensity of photosimulation of inhibitory pre-BötC neurons in slices did not result in a reduction or termination of the activity of VGAT-positive inspiratory neurons by a possible activation of some other (non-inspiratory) inhibitory neurons located within the pre-BötC (e.g., such as the pre-I_pBC_ in our model) at higher stimulation intensities (as seen in Fig 3C and 3D), as could be expected considering our data obtained *in situ* (Figs 4E,4F and 5B–5D), as well as the proposed network interactions in the model (see Fig 9B) and the results of our simulations (see Figs 10E,10F and 11B–11D). Our explanation for this lack of inspiratory activity termination *in vitro* is that, in contrast to the more intact preparations, slices operate in very different physiological conditions engaging different rhythmogenic mechanisms [3,4,6,8,51,52]. Moreover, these slice preparations contain highly reduced respiratory circuitry, lacking many important structures, such as pontine circuits, which in the more intact preparations (*in situ* and *in vivo*) are known to provide excitatory drive to, and increased excitability of, post-inspiratory neurons [3,4,6,29,53–55]. Based on the results of our previous modeling studies [4,6,10], all post-I neurons in such conditions (without additional excitatory drives from the pons and other structures not present in the slice) remain silent and cannot escape from inhibition provided by the early-I type inspiratory inhibitory neurons during applied photostimulation.

To verify site-specificity of perturbations in the pre-BötC or BötC regions targeted for optical activation of VGAT-ChR2 expressing inhibitory neurons *in situ*, we systematically repositioned the optical cannula bilaterally in reticular formation sites outside of these regions. Laser illumination (2–5 mW) in the rVRG immediately (~ 200 μm) caudal to the pre-BötC region did not cause optical perturbations of the respiratory rhythm, consistent with the coordinates we used for bilateral positioning the optical cannulae in the pre-BötC from our extracellular recordings of respiratory neuron activity within the ventral respiratory column in the *in situ* experimental preparation. We accordingly positioned the cannulae more rostrally at our electrophysiologically established coordinates with predominant post-I and aug-E neuron activity to target the BötC. Furthermore, the perturbations produced by photostimulation at low laser intensities (≤ 2.0 mW) where light scattering is reduced in the pre-BötC and BötC sites targeted were opposite, with augmentation of the inspiratory frequency with photostimulation in the pre-BötC region and inhibition of the rhythm with BötC regional photostimulation, indicating site-specificity of the perturbations.

### The role of inhibitory interactions within the pre-BötC and between the pre-BötC and BötC for respiratory rhythm and pattern generation

There has been a long-lasting debate concerning the role of the BötC and inhibitory circuit interactions within and between the pre-BötC and BötC for respiratory rhythm and pattern generation [2–4,6–8,42–46,56]. In this study, we have shown that selective optical activation of inhibitory neurons within either the pre-BötC or the BötC can terminate inspiration and reset the rhythm after a latency that according to our model may be defined by the activity of the inhibitory expiratory (aug-E) neurons located in the BötC. Our model also suggests that the post-inspiratory period insensitive to activation of all pre-BötC neurons [47] depends on the activity of post-I neurons in the BötC. Hence both types of inhibitory neurons in the BötC (post-I and aug-E) as well as the post-I_pBC_ population in the pre-BötC could be specific targets of different inputs controlling breathing and, therefore, these populations may play an important role in respiratory pattern generation and control under various behavioral conditions.

### Different types of post-I neurons and their location in the pre-BötC/BötC core network

In previous compartmental models of the pre-BötC/BötC respiratory core network only one type of inhibitory post-I neurons was usually considered (whether these neurons were called post-I or E-Dec or dec-E). The population of these neurons was assumed to be located within the BötC [3–8,10,11], since this compartment does contain a larger number of neurons with post-inspiratory activity (e.g. [23,25,57]). In the present model, we incorporated two different types of post-I neuron populations: a BötC post-I population with long decrementing activity during expiration and a post-I_pBC_ with short transient post-inspiratory activity (see Fig 9B and 9C). There are two issues here that require a more careful consideration and discussion.

The first issue concerns the heterogeneity of the post-I neuron population. Two types of neurons with post-I activity patterns have been described: the post-I neurons of so-called E-DEC (or dec-E) type with activity decrementing over the entire, or a large part of, expiration [23,25,57–63], and the post-I neurons with very short or transient firing during the first half of expiration (during the post-inspiratory phase) that were first described by Diethelm Richter [1,64]. The debated issue has been whether these two types of post-I neurons represent functionally the same or distinct neuron populations [23,25,65]. This issue remained unresolved although both types of these neurons were recorded, often in the same experiments (e.g., [26,66,67]) and some earlier models included these two post-I neuron types (dec-E and post-I) as separate populations to perform different roles in the network [65]. Taking into account previous evidence and experimental and modeling data presented here, it is likely that post-I is not a single, functionally unique neuron population, but rather multiple (at least two, as in our model) populations that are involved in termination of inspiration or activated right after inspiration and have short or long lasting decrementing activity, including recently discovered post-I neurons with intrinsic bursting properties [68,69]. These different post-I populations may mediate different inputs (e.g. from the pons and from NTS) and/or contribute to termination of inspiration during different behavioral processes (e.g., during eating, drinking, swallowing, vocalization, etc.).

The second issue concerns the location of neurons exhibiting post-I activity. Even though the majority of these neurons are located within the BötC [23,25,57], different types of post-I/E-DEC neurons are widely distributed over the ventral respiratory column [9,70] and are present in the pre-BötC [26]. Moreover, based on some earlier investigations, the post-I neurons with short firing patterns are located more rostrally in the area that likely corresponds to the pre-BötC [25,66,71], which is consistent with the model presented here.

### Comparison with other studies utilizing optogenetic perturbations of respiratory populations in the pre-BötC or BötC

Selective optogenetic activation of inhibitory (glycinergic only) neurons in the BötC was previously performed by Sherman et al. [46]. In their *in vivo* experiments in mice, optical stimulations applied during inspiration always terminated inspiratory activity, whereas stimulations applied during the expiratory phase delayed the onset of the next inspiration. Sustained stimulations of glycinergic pre-BötC neurons produced apnea. At first glance, these results look contradictory to our results, but can be reconciled with our data in the following way.

The *in vivo* preparations used by Sherman et al. [46] exhibited a respiratory rhythm that was considerably faster (~ 500 ms cycle period) than what is reported here (~ 2 s) for *in situ* preparations. They, however, reported a similar fixed latency between the end of the photostimulus and the beginning of the subsequent inspiratory burst (~ 300 ms). Taking into account an oscillation period of ~ 500 ms (~ 300 ms of expiratory phase) and ~ 100 ms stimulus duration, this would naturally result in a phase delay (which is what they reported) and not in a phase advance which we found for the much slower respiratory rhythm in our experiments. This is further corroborated by the delay of inspiration in our experiments when stimuli were applied at the end of expiration (see Figs 4D and 10D). Thus, in both Sherman et al. [46] and our experiments, the underlying mechanism can be a rebound activation of inspiration after a fixed latency that is independent of the respiratory frequency.

Further, Sherman et al. [46] reported that optogenetic stimulation of glycinergic neurons terminated inspiration, which is consistent with our data and model simulations for high stimulation intensities (laser power ≥ 2.0 mW in our experiments). The same is true for strong sustained photostimulation, where Sherman et al. [46] reported apnea for the duration of the stimulation (Figs 5C and 11C).

Biphasic responses to optical stimulations depending on laser intensity (as those we observed) could be absent in the study by Sherman et al. [46] because: (1) they only used “strong” light stimulations corresponding to ≥ 2.0 mW in our experiments; (2) they used viral transfection to express ChR2 which might lead to stronger expression and thus a stronger optogenetic excitation of neurons; or (3) the selective targeting of glycinergic neurons might change the balance of activation of specific populations depending on the transmitter phenotype composition in each respiratory population. For example, if the population that we labeled as post-I_pBC_ were predominantly glycinergic while other populations have a higher proportion of GABAergic inhibitory neurons, then our model prediction would be very similar to the results shown by Sherman et al. [46] even under lower intensity stimulations.

Interestingly, single unit recordings from various types of pre-BötC neurons in the Sherman et al. [46] study showed inhibition of these neurons during photostimulation, although there should be some inhibitory (glycinergic) neurons expressing ChR2 within pre-BötC that were activated by light to inhibit all other neurons. Our modeling studies suggest that the neurons active during optogenetic stimulation should be a population of post-I neurons located in the pre-BötC (post-I_pBC_) which strongly inhibits all other pre-BötC neurons (Fig 10D–10F). The above analyses further support the important role of inhibition within the pre-BötC and between the pre-BötC and BötC.

In a recent modeling study, Oku and Hülsmann [72] also analyzed the results published by Sherman et al. [46] using a computational model that reproduced the neuronal composition and connectivity within and between the pre-BötC and BötC from our previous models [3,4,6–8,10]. In line with our modeling studies, they concluded that sole activation of the inhibitory early-I population in the pre-BötC (called I-DEC) could not reproduce the Sherman et al. [46] results. They, however, did not add an additional post-I population to the pre-BötC as we did, but simply assumed that in Sherman et al. [46] experiments, glycinergic BötC neurons were also activated by light (in addition to those in the pre-BötC) due to wider light scattering and/or spreading of the virus driving expression of ChR2 into the BötC. The latter could also explain the lack of extracellular recordings from pre-BötC neurons activated by light stimulation in Sherman et al. [46] (see above). In addition, the model of Oku and Hülsmann [72] could reproduce the termination of inspiration and rebound activation of the next inspiration by stimulating both pre-BötC and BötC inhibitory populations. However, their model did not reproduce the fixed latency before the inspiration onset observed in both Sherman et al. [46] and our present study.

As described in Results, Alsahafi et al. [47] used site-specific optogenetic stimulation of pre-BötC and BötC neurons *in vivo*. They stimulated both inhibitory and excitatory neurons due to the viral vector construct with a general neuronal promoter (synapsin) employed. To test the ability of our model to reproduce their results, we used our model without adjusting any of the model parameters that were used to reproduce our data. As is, the model was able to reproduce many of their results, assuming simultaneous activation of inhibitory and excitatory neuron populations. Specifically: (1) sustained stimulation of the pre-BötC (all pre-BötC populations in the model) increased respiratory frequency (Fig 14A), (2) respiratory activity could be entrained 1:1 by repetitive short light pulses activating the pre-BötC (Fig 14B), (3) sustained activation of the BötC (all populations in the model) produced apnea (Fig 11), and (4) short phase-dependent simulations reset the respiratory rhythm except for a short post-inspiratory period during which the rhythm could not be reset (Fig 15). These simulations provided an additional validation of our model and further evidence for the concept that the BötC and the inhibitory interactions within and between the pre-BötC and BötC play a significant role in respiratory rhythm and pattern generation and control.

### Latency of rebound activation

Sufficiently strong optogenetic stimulation of inhibitory neurons either within the pre-BötC or the BötC suppressed rhythmic activity in the network for the duration of the stimulus and elicited a rebound activation of inspiration with a fixed latency of about 300 ms after the end of the stimulus (Figs 4 and 6). As discussed above, a similar latency has been reported for the photoactivation of glycinergic neurons in the pre-BötC by Sherman et al. [46]. Our modeling studies suggest that one possible explanation for this constant latency between the end of the photostimulation and the onset of the next inspiratory burst is a transient activation of the aug-E population and subsequent rebound activation of the pre-I/I population. An alternative explanation could be a delayed activation after inhibition of the pre-I/I population that is inherent to the pre-I/I population itself. Both options could be tested in future studies by either recording from aug-E neurons during photostimulation of the inhibitory pre-BötC neurons or by optogenetically inhibiting the excitatory pre-I/I population directly and analyzing rebound characteristics of this population.

### Limitations of our experimental and modeling studies

The main limitation of our experimental study is a lack of cellular-level recordings from all of the different neuron types within the pre-BötC or the BötC during different site- and respiratory phase-specific photostimulations. In the present studies, we have assessed from simultaneous extracellular recordings the perturbations of pre-BötC inspiratory population activity at the level of the inspiratory oscillator. This included our recordings of this activity during photostimulation of the BötC region, which reliably caused inhibition of the pre-BötC neurons, consistent with inhibitory input connections from the BötC. Our model simulations make specific predictions about activity perturbations of the different types of pre-BötC and BötC neurons and it remains an important problem to compare these predictions to neuronal activity recordings to further test the validity of the circuit interactions represented by the model.

We also note that our transgenic approach with the VGAT promoter-driven expression of ChR2 in all inhibitory neurons did not allow us to separately investigate the roles of GABAergic and glycinergic neuron populations. We have previously proposed more complex model circuit configurations that make functional distinctions between GABAergic and glycinergic populations (e.g., [43]), and while there is a large population of neurons co-expressing GABA and glycine (e.g.,[21]), it will be of considerable interest to employ transgenic approaches allowing selective manipulations of GABAergic or glycinergic neuron activity to possibly differentiate their functional roles in respiratory pattern generation. Moreover, additional tests of the inhibitory neuron circuit architecture and function that we propose should also be done using regional photoinhibition to assess activity perturbations associated with attenuation/loss of inhibitory circuit function. Some comparisons have been performed for neuronal activity recording data from studies where glycinergic postsynaptic inhibition was pharmacologically blocked [43]. It is likely that experiments utilizing VGAT promotor-driven expression of inhibitory opsins, or expression driven by other promoters (e.g., GlyT2 to target glycinergic neurons [46]), will provide new insights.

There are certain limitations in the current modeling approach. Specifically, in contrast to some of our previous models [3,4,6,7], we used a simplified description of neural population activity, which has computational and conceptual advantages [10,11], but did not allow us to consider in detail the roles of neuronal recruitment and synchronization in each population under different conditions. This modeling approach also did not allow us to investigate the potential roles of intrinsic neuronal properties, known to be present in the respiratory neurons, such as different calcium, calcium-dependent potassium, and calcium-activated non-selective cationic currents, that are present in different respiratory neurons [14,15,73–76]. These issues can ultimately be addressed by implementing populations of neurons described by more detailed conductance-based models to represent the interacting populations within the respiratory CPG core circuits that we have proposed in the present study.

## Methods

### Ethics statement

All animal procedures were approved by the Animal Care and Use Committee of the National Institute of Neurological Disorders and Stroke.

### Cre-dependent double and triple transgenic (Tg) mouse experimental models

For our optogenetic experiments photostimulating inhibitory neurons, we used Tg mice expressing Channelrhodopsin-2 (ChR2) and reporter fluorescent proteins in glycinergic and GABAergic neurons driven by Cre recombinase expressed in these neurons under control of the vesicular GABA transporter (VGAT) promoter [77–79]. To produce this mouse line, we first crossed a Slc32a1^tm2(Cre)Lowl^ knock-in Cre-driver mouse line (VGAT-ires-Cre, the Jackson Laboratory, Bar Harbor, ME) with a Cre-dependent reporter mouse strain [B6;Cg-Gt(ROSA)26Sor^tm9(CAG-tdTomato)Hze^, Rosa-CAG-LSL-tdTomato-WPRE, the Jackson Laboratory] to obtain offspring expressing red fluorescent protein variant tdTomato in Cre-expressing VGAT-positive neurons. This double Tg line (VGAT-tdTomato) was analyzed histologically to verify tdTomato expression in glycinergic and GABAergic neurons labeled by antibodies (below), and subsequently crossed with a Cre-dependent optogenetic mouse strain [B6;129S-Gt(ROSA)26Sor^tm32(CAG-COP4*H134R/EYFP)Hze^, Rosa-CAG-LSL-ChR2(H134R)-EYFP-WPRE, the Jackson Laboratory] to obtain a triple Tg mouse line (VGAT-tdTomato-ChR2-EYFP), in which Channelrhodopsin-2 (ChR2)-EYFP fusion proteins are specifically expressed in VGAT-Cre positive neurons.

### Immunohistochemical labeling of pre-BötC neurons

Fluorescence immuno-labeling with glycine and GABA antibodies was used to verify glycine and GABA expression in VGAT-tdTomato neurons within the pre-BötC and BötC in immersion or transcardially perfusion fixed brainstem tissue sections from neonatal and mature VGAT-tdTomato double Tg mouse lines. The medulla oblongata used for histological analyses was fixed in 4% paraformaldehyde (wt/vol) in phosphate-saline buffer, and cryoprotected overnight at 4°C in 30% sucrose, 0.1 M PBS solution and sectioned coronally or parasagittally at 30 or 50 μm with a freezing microtome. For fluorescence immunohistochemistry, floating sections were incubated with 10% donkey serum in PBS with Triton X-100 (0.3%) and subsequently incubated for 48–72 hours at room temperature with primary antibodies for GABA (chicken anti-GABA, 1:300, ab62669, Abcam, Cambridge, MA), for glycine (rabbit anti-glycine, 1:5000, IG1001, ImmunoSolution; MP Biomedicals, Solon, OH), and also for choline acetyltransferase (ChAT) (goat anti-ChAT, 1:200, AB144P, EMD Millipore, Billercia, MA) to label motoneurons. Individual sections were then rinsed with PBS and incubated for 2 hours with secondary antibodies (donkey anti-rabbit Alexa Fluor 647, 1:500 for glycine labeling; donkey anti-chicken Alexa Fluor 647, 1:500 for GABA labeling; donkey anti-goat Alexa Fluor 488, 1:500 for ChAT, Jackson ImmunoResearch, West Grove, PA). Individual sections were mounted on slides and covered with an anti-fading medium (Fluoro-Gel; Electron Microscopy Sciences, Hatfield, PA). Fluorescent labeling of neurons was visualized with a laser-scanning confocal imaging system (LSM 510, Zeiss, Oberkochen, Germany) or two-photon microscopy (TCS SP5 II MP, Leica, Buffalo Grove, IL). All images were color/contrast enhanced and adjusted with a thresholding filter in Adobe Photoshop.

We validated specificity of the glycine and GABA antibodies used by analyzing antibody labeling in transgenic (Tg) mouse lines, in which fluorescent proteins (tdTomato or GFP) are expressed in excitatory or inhibitory neurons by the cell type-specific promoters VgluT2 for glutamatergic, GlyT2 for glycinergic, and GAD67 for GABAergic neurons. We verified glycine antibody co-labeling in all of the GlyT2-tdTomato positive glycinergic neurons within the pre-BötC and BötC regions in our GlyT2-tdTomato Tg mouse line, which was obtained by crossing a GlyT2-Cre line [B6.FVB(cg)-Tg(Slc6a5-cre)KF109Gsat/Mmucd, MMRRC, UC Davis] with a Cre-dependent tdTomato reporter strain [B6.Cg-Gt(ROSA)26Sor^tm9(CAG-tdTomato)Hze^/J, Jackson Laboratories]. GlyT2 has been regularly used as a specific promotor for glycinergic neurons in the field (e.g., [19,46]). We also verified GABA antibody co-labeling in all of the GAD-67 positive GABAergic neurons, but no labeling in VgluT2-tdTomato positive neurons within the pre-BötC and BötC regions in the triple Tg mouse line (VgluT2-tdTomato-GAD67-GFP), which was obtained by crossing the VgluT2-tdTomato mouse line with a GAD67-GFP knock-in mouse line ([80]) as we have previously reported ([81]). In this Tg mouse line, we also verified no glycine antibody labeling in VgluT2-tdTomato positive neurons ([81]). In addition, we verified the same labeling patterns of the pre-BötC and BötC neurons with different GABA antibodies [i.e., chicken anti-GABA ab62669, Abcam reported in this study, and rabbit anti-GABA A2052, Sigma (e.g., [21,82])]. We also note that the VGAT Cre-driver mouse line used in our study (VGAT-ires-Cre, the Jackson Laboratory) has been validated with *in situ* hybridization analyses for VGAT mRNA, which has verified that Cre activity is expressed in all of VGAT mRNA-positive neurons analyzed ([83]).

### Rhythmically active medullary slice preparations *in vitro*

We performed combined optogenetic stimulation and whole-cell patch-clamp recordings with rhythmically active *in vitro* slice preparations (300–400 μm thick) of the medulla oblongata that were cut from neonatal [postnatal day 3 (P3) to P8] triple Tg mouse line (VGAT-tdTomato-ChR2-EYFP) of either sex (Fig 3A). These slices contain the active bilateral pre-BötC and rostral end of the hypoglossal motor nucleus (XII) with intact XII nerve rootlets for recording inspiratory motor output ([21]). The slice was superfused (4 ml/min) in a recording chamber (0.2 ml) mounted on the stage of an upright laser scanning microscope with artificial cerebrospinal fluid (ACSF) containing the following (in mM): 124 NaCl, 25 NaHCO_3_, 3 KCl, 1.5 CaCl_2_, 1.0 MgSO_4_, 0.5 NaH_2_PO_4_, 30 D-glucose equilibrated with 95% O_2_ and 5% CO_2_ (pH = 7.35–7.40 at 27°C). During experiments, rhythmic respiratory network activity was maintained by elevating the superfusate solution K^+^ concentration to 8–9 mM.

Electrophysiological signals recorded from XII nerve rootlets with fire-polished glass suction electrodes (50–100 μm inner diameter) were amplified (50,000–100,000X; CyberAmp 380, Molecular Devices Sunnyvale, CA), band-pass filtered (0.3–2 kHz), digitized (10 kHz) with an AD converter (PowerLab, AD Instruments, Inc., Colorado Springs, CO), and then rectified and integrated digitally with Chart software (AD Instruments). Whole-cell current-clamp data were recorded with an EPC-9 patch-clamp amplifier (HEKA Electronics Inc., Mahone Bay, Nova Scotia, Canada) controlled by PatchMaster software (HEKA; 2.9 kHz low-pass filter, sampled at 100 kHz). Whole-cell recording electrodes (borosilicate glass pipette, 4–6 MΩ), positioned with 3-dimensional micromanipulator (Scientifica), contained the following (in mM): 130.0 K-gluconate, 5.0 Na-gluconate, 3.0 NaCl, 10.0 HEPES, 4.0 Mg-ATP, 0.3 Na-GTP, and 4.0 sodium phosphocreatine, pH 7.3 adjusted with KOH. In all cases, measured potentials were corrected for the liquid junction potential (-10 mV). Series resistance was compensated on-line by 80%, and the compensation was periodically readjusted.

Optical live imaging of tdTomato-EYFP labeled neurons in the slices was performed with a Leica multi-photon laser scanning upright microscope (TCS SP5 II MP with DM6000 CFS system, a 20X water-immersion objective, N.A. 1.0, Leica). A two-photon Ti:Sapphire pulsed laser (MaiTai, Spectra Physics, Mountain View, CA) was used at 800–880 nm with DeepSee predispersion compensation.

### Arterially perfused mouse brainstem-spinal cord preparations *in situ*

**Fig 16:**
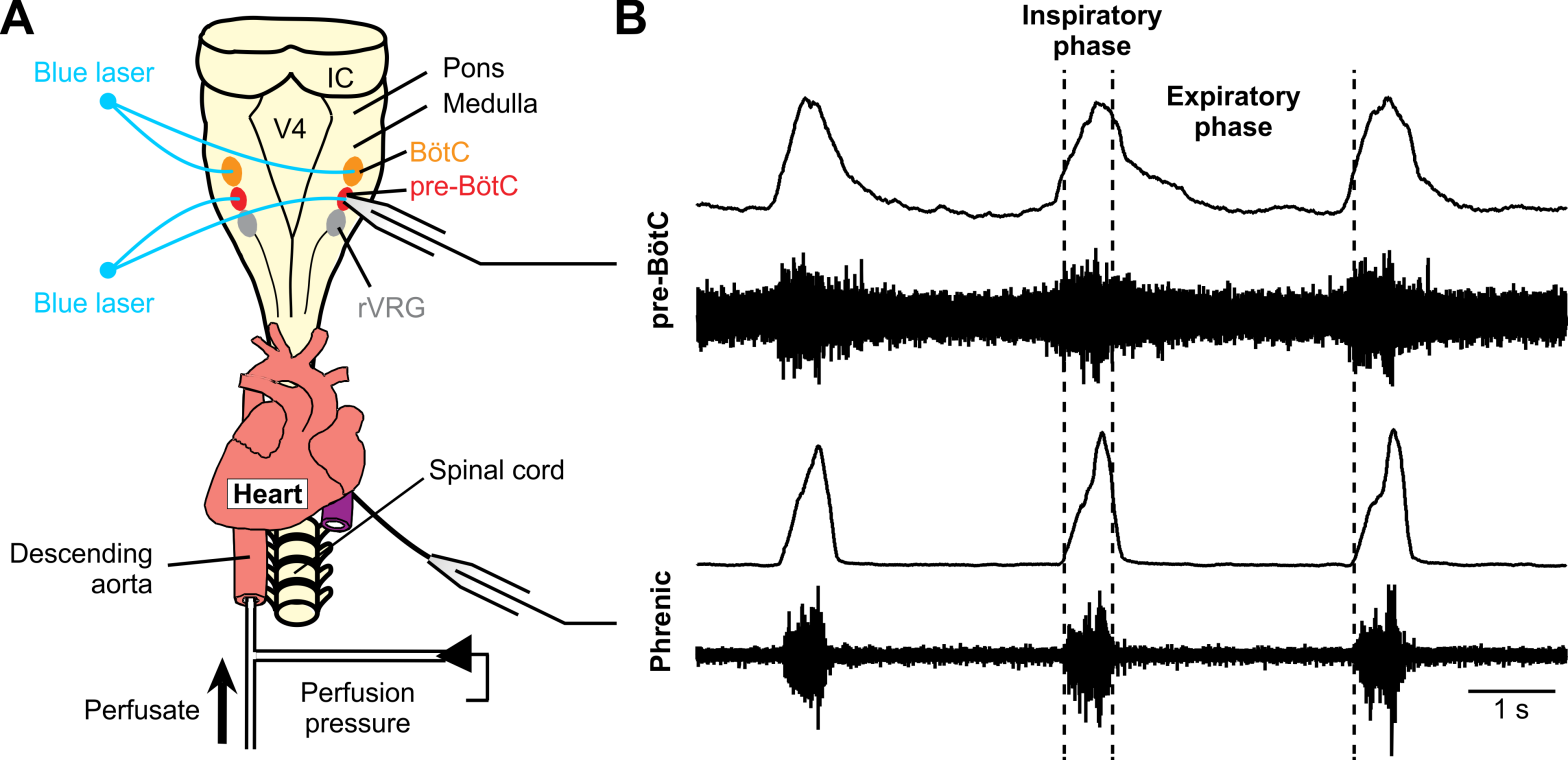
Experimental setup for site-specific optogenetic stimulation of inhibitory neurons within the pre-BötC or BötC. (**A**) Dorsal view of the *in situ* arterially perfused transgenic mouse brainstem-spinal cord preparation. (**B**) Representative examples of extracellular recordings of pre-I/I population activity in the pre-BötC and inspiratory activity recorded from the phrenic nerve. Each panel shows raw (lower trace) and integrated (upper trace) pre-BötC and PN nerve inspiratory discharge. Abbreviations: V4, 4th ventricle; IC, inferior colliculus; pre-BötC, pre-Bötzinger complex; BötC, Bötzinger complex; rVRG, rostral ventrolateral respiratory group.

Combined photostimulation and electrophysiological recording experiments were performed with *in situ* arterially perfused brainstem-spinal cord preparations (Fig 16A) from the mature triple Tg mouse line (VGAT-tdTomato-ChR2-EYFP) of either sex (20 – 30g, 2–4 months old), in which complex cellular and circuit interactions involving the pons, BötC, pre-BötC and rVRG generate a normal 3-phase rhythmic respiratory neural activity pattern [6,84]. Preheparinized (1,000 units, given intraperitoneally) mice were anaesthetized deeply with 5% isoflurane until loss of the paw withdrawal reflex, and the portion of the body caudal to the diaphragm was removed. The head and thorax were immersed in ice-chilled carbogenated ACSF solution containing the following (in mM): 1.25 MgSO_4,_ 1.25 KH_2_PO_4_, 5.0 KCl, 25 NaHCO_3_, 125 NaCl, 2.5 CaCl_2_, 10 dextrose, 0.1785 polyethylene glycol. The brain was decerebrated at a precollicular level, and the descending aorta, thoracic phrenic nerve and cervical vagus nerves were surgically isolated. The dorsal brainstem was exposed by craniotomy and cerebellectomy. The preparation was transferred to a recording chamber and secured in a stereotaxic head frame with dorsal side up. The descending aorta was cannulated with a double lumen catheter (DLR-4, Braintree Scientific, Braintree, MA) for ACSF perfusion with a peristaltic roller pump (505D, Watson-Marlow, Wilmington, MA) and for recording of perfusion pressure with a pressure transducer. The ACSF perfusate was gassed with 95% O2-5% CO2 and maintained at 31°C. Rocuronium bromide (2–4 μg/ml; SUN Pharmaceutical Industries, Cranbury, NJ) was added to the perfusate to block neuromuscular transmission. Throughout the experiments, the perfusion pressure was maintained between 60 and 80 mmHg with vasopressin application (200–400 pM as required; APP Pharmaceuticals, Los Angeles, CA) and by adjusting the perfusion pump speed to avoid the possible effects of pressure changes on respiratory activity.

To monitor respiratory network activity and motor output, we recorded inspiratory activity from phrenic nerves (PN) and/or in some cases extracellular population activity from pre-BötC inspiratory neurons as described previously [81] that allowed us to directly analyze activity perturbations at the level of the inspiratory rhythm generator. We also recorded extracellular population activity from BötC post-I or aug-E type of expiratory neurons to verify photostimulation of these BötC inhibitory neurons by laser illumination in the BötC region. Extracellular population activity from the pre-BötC and BötC (Figs 8 and 16) was recorded by a dorsal approach with a fine-tipped glass pipette (3–5 MΩ resistance) filled with 0.5 M sodium acetate (Sigma) positioned by a computer-controlled 3-dimensional micromanipulator (MC2000, Märzhäuser) based on our established anatomical coordinates for the pre-BötC and BötC [81]. Electrophysiological signals were amplified (50,000–100,000X, CyberAmp 380, Molecular Devices, Sunnyvale, CA), band-pass filtered (0.3–2 kHz), digitized (10 kHz) with an AD converter [Cambridge Electronics Design (CED), Cambridge, UK], and then rectified and integrated digitally with Spike2 software (CED).

### Photostimulation of inhibitory pre-BötC or BötC neuronal populations

Laser illumination for photostimulation experiments was performed with a blue laser (473 nm; OptoDuet Laser, IkeCool, Los Angeles, CA), and laser power (0.5–5.0 mW) was measured with an optical power and energy meter (PM100D, ThorLabs, Newton, NJ). Illumination epochs were controlled by a pulse stimulator (Master-8, A.M.P.I., Jerusalem, Israel). To apply the laser during the specific respiratory phase (i.e., I-phase or E-phase), the onsets of inspiratory phrenic activity were detected as the times the integrated PN signals crossed a defined threshold (normally 20% of the peak amplitude), and photostimulation epochs controlled by computer-triggered TTL pulses were delivered after pre-programmed delays from the detected onset of PN activity. Optical fiber(s), from a bifurcated fiberoptic patch cable, each terminated by an optical cannula (100 μm diameter, ThorLabs) were positioned unilaterally on the surface of the pre-BötC in the *in vitro* rhythmically active slice preparations (Fig 3A), and implanted bilaterally to a depth immediately dorsal to the pre-BötC or BötC in the brainstem-spinal cord preparations *in situ* (Fig 16A). Positioning of the optical fibers by micromanipulators was based on coordinates for the pre-BötC and BötC from our extracellular recordings of respiratory neurons within the ventral respiratory column in the present and previous combined electrophysiological and optogenetics-based studies [81]. We conducted control experiments with *in situ* preparations from the VGAT-tdTomato (non-ChR2-expressing) Tg mouse strains (n = 2), and found that no light-induced perturbations of the respiratory frequency or burst amplitude of inspiratory PN activity with the optical cannula positioned bilaterally in the pre-BötC or the BötC.

### Signal analysis of respiratory parameters

All digitized electrophysiological signals were analyzed by automated procedures to extract respiratory parameters from integrated PN or pre-BötC inspiratory activities, performed with Matlab (R2016a) and Python (3.5) software utilizing the computational resources of the NIH HPC Biowulf cluster. Inspiratory events were detected from the smoothed integrated PN signals via a 200 ms window moving average and peak detection algorithm based on detecting ridges in the continuous wavelet transform with appropriate peak width boundaries for each recording. Following peak detection, interburst interval was computed to obtain the respiratory frequency. Inspiratory amplitude was calculated as the difference between peak value and the local baseline voltage, which was calculated as the value corresponding to the peak of the Gaussian kernel density estimation in a local 10 second window.

Latencies were calculated as the time difference between the end of the light stimulus and the onset of the next PN inspiratory burst, detected by the integrated PN signals crossing the threshold at 20% of the peak amplitude as described above.

For statistical analysis (significant p-value < 0.05), experimental conditions were compared with a one sample Student’s t-test. Summary data are presented as means ± SEM.

### Model description

The schematic of the model of the core pre-BötC and BötC microcircuits developed in this study is shown in Fig 9B. The model is based on network interactions within pre-BötC and BötC proposed in a series of previous models [3–8,10,11] and specifically represents an extension of the model of Rubin et al. [10] (Fig 9A). Synaptic interactions between neuron populations included in that earlier model had been justified in previous publications [3,4,6]. To reproduce the experimental data presented in this study we extended the Rubin et al. [10] model (Fig 9A) by incorporating an additional population of post-inspiratory inhibitory neurons in the pre-BötC (post-I_pBC_, Fig 9B). The reasons for including this additional post-I population are described in the Results section and further justification for the presence of two separate post-I populations, including the one within the pre-BötC, is presented in the Discussion. All connections of the incorporated post-I_pBC_ population were implemented the same as the connections of the post-I population in the BötC. Weights of synaptic connections in the present model have been modified from the original Rubin et al. [10] model to match our experimental data and/or to accommodate the inclusion of the additional post-I_pBC_, population but not altered otherwise.

The model includes five neuron populations (*i* = 1, 2, 3, 4, 5), three of which are located in the pre-BötC (the excitatory pre-inspiratory, pre-I/I, *i* = 1, the inhibitory early-inspiratory, early-I, *i* = 2, and the novel inhibitory post-I_pBC_, *i* = 5) and two inhibitory populations in the BötC (the augmenting-expiratory, aug-E, *i* = 3, and the post-I, *i* = 4) (Fig 9B).

Each neural population is described by an activity-based model, in which the dependent variable *V*_*i*_ represents an average voltage for population *i* and each output *f*_*i*_*(V*_*i*_*)* (0 ≤ *f*_*i*_*(V*_*i*_*)* ≤ 1) represents the average or integrated population activity at the corresponding average voltage [10,85]. This description allows an explicit representation of ionic currents, specifically of the persistent (slowly inactivating) sodium current *I*_*NaP*_ [10] responsible for state-dependent generation of intrinsic bursting activity in the pre-BötC [86–88]. Because we consider regimes in which neurons within each population switch between silence and active spiking in a generally synchronized way, we assume that the dynamics of the average voltages in the model can be represented by a conductance-based framework but without fast membrane currents responsible for spiking activity, which tremendously simplifies the model description and analysis [10]. While such a simplified activity-based description of neuron populations allows for the qualitative reproduction of experimental data, it does not imply an exact correspondence between experimental and modeling results with regard to single neuron activity and synaptic interactions.

The membrane potential (*V*_*1*_) of the excitatory pre-I/I population (*i* = 1) with *I*_*NaP*_-dependent intrinsic oscillatory properties obeys the following differential equation:

$$C∙\frac{{dV}_{1}}{dt}=-I_{NaP}{-I}_{K}-I_{L_{1}}-I_{{SynE}_{1}}-I_{{SynI}_{1}}-I_{{ChR}_{1}},$$ (1)

where *I*_*K*_ represents the potassium delayed rectifier current included in the pre-I/I population.

The other four populations do not have *I*_*NaP*_ and *I*_*K*,_ but have adaptive properties defined by the outward potassium currents $I_{{AD}_{i}}$. For each of these populations (*i* ∈ {2,3,4,5}), the average membrane potential is described as:

$$C∙\frac{{dV}_{i}}{dt}=-I_{AD_{i}}-I_{L_{i}}-I_{{SynE}_{i}}-I_{{SynI}_{i}}-I_{{ChR}_{i}}.$$ (2)

In Eqs (1) and (2), *C* is the neuronal membrane capacitance; $I_{L_{i}}$ is the leak current; $I_{{SynE}_{i}}$ and $I_{{SynI}_{i}}$ are the excitatory and inhibitory synaptic currents, respectively, and $I_{{ChR}_{i}}$ is the excitatory ChR2 current. The currents are described as follows:

$$I_{NaP}={\bar{g}}_{NaP}{∙m}_{NaP}{∙h}_{NaP}∙(V_{1}-E_{Na});$$

$$I_{K}={\bar{g}}_{K}∙m_{k}^{4}{∙h}_{NaP}(V_{1}-E_{K});$$

$$I_{{AD}_{i}}={\bar{g}}_{AD}{∙m}_{{AD}_{i}}∙(V_{i}-E_{K});$$

$$I_{L_{i}}={\bar{g}}_{L}∙(V_{i}-E_{L});$$

$$I_{{SynE}_{i}}={\bar{g}}_{SynE}∙{drive}_{i}∙(V_{i}-E_{SynE}),\;for\;i\neq 2;$$

$$I_{{SynE}_{2}}={\bar{g}}_{SynE}∙\left({drive}_{2}+a_{12}{∙f}_{1}(V_{1})\right)∙\left(V_{2}-E_{SynE}\right);$$

$$I_{{SynI}_{i}}={\bar{g}}_{SynI}∙\sum_{\begin{array}{c} j=2\\ j\neq i \end{array}}^{5}b_{ji}{∙f}_{j}(V_{j})∙(V_{i}-E_{SynI});$$

$$I_{{ChR}_{i}}={\bar{g}}_{{ChR}_{i}}{∙stim}_{{ChR}_{i}}(t)∙(V_{i}-E_{ChR}),$$ (3)

where *I*_*X*_ is the current, ${\bar{g}}_{X}$ is the maximal conductance and *E*_*X*_ is the reversal potential of the corresponding channel *x*; *a*_12_ defines the weight of the excitatory synaptic input from the pre-I/I to the early-I population; *b*_*ji*_ defines the weight of the inhibitory input from population *j* to population *i* (*i* ∈ {1,2,3,4,5}, *j* ∈ {2,3,4,5}) (Fig 9B); *drive*_*i*_ defines the excitatory drive to population *i*; *stim*_*ChRx*_(*t*) represents the laser intensity of the ChR2 stimulation.

The nonlinear function *f*_*i*_(*V*_*i*_) defines the output activity of each population unit (indirectly representing the rate of spiking activity)

$$f_{i}(V_{i})=\left\{ \begin{array}{c} 1/(1+exp[-(V_{i}-V_{1/2})/k_{V_{i}}]),\;if\;V_{i}\geq -60mV;\\ 0,\;if\;V_{i}<-60mV, \end{array}\right.$$ (4)

where *V*_1/2_ is the half-activation voltage and $k_{V_{i}}$ defines the slope of the output function for each population *i*.

Two types of slow variables are implemented in the model. One variable (*h*_*NaP*_) represents the slow inactivation of the persistent sodium current [86] in the pre-I/I population; the other variables ($m_{{AD}_{i}},\;i\in \{2,3,4,5\}$) describe the level of adaptation in the other populations:

$$\tau_{hNaP}(V_{1})∙\frac{d}{dt}h_{NaP}=h_{\infty NaP}(V_{1})-h_{NaP},$$

$$\tau_{{AD}_{i}}∙\frac{d}{dt}m_{{AD}_{i}}=k_{{AD}_{i}}{∙f}_{i}(V_{i})-m_{{AD}_{i}}.$$ (5)

Voltage dependent activation and inactivation variables and time constants for the persistent sodium and potassium rectifier channels in pre-I/I population are described as:

$$m_{NaP}=1/\left(1+exp[\left(-V_{1}+40\right)/6]\right);$$

$$h_{\infty NaP}=1/\left(1+exp[\left(V_{1}+48\right)/6]\right);$$

$$\tau_{hNaP}=\tau_{hNaPmax}/cosh[\left(V_{1}+48\right)/12];$$

$$m_{K}=1/\left(1+exp[\left(-V_{1}+30\right)/4]\right).$$ (6)

### Model parameters

Model parameters were not based on direct experimental measurements, but adapted from the model of Rubin et al. [10] and other previous models [38,40,89] and modified/adjusted to accommodate the incorporation of the additional post-I_pBC_ population. These adjustments were done carefully to allow, on one hand, reproduction of major features of the previous models (and related experimental data) and, on the other hand, to qualitatively reproduce the experimental data presented here.

The following parameter values were used:

- Membrane capacitance (pF): *C* = 20.
- Maximal conductances (nS): *g*_*NaP*_ = 5.0, *g*_*K*_ = 5.0, *g*_*AD*_ = 10.0, *g*_*L*_ = 2.8, *g*_*SynE*_ = 10.0, *g*_*SynI*_ = 60, *g*_*ChR*_ = 8_._
- Reversal potentials (mV): *E*_*Na*_ = 50, *E*_*K*_ = –85, *E*_*L*_ = –60, *E*_*SynE*_ = 0, *E*_*SynI*_ = –75, *E*_*ChR*_ = 0.
- Synaptic weights: *a*_*12*_ = 0.5; *b*_*21*_ = 0, *b*_*23*_ = 0.37, *b*_*24*_ = 0.26, *b*_*25*_ = 0.27; *b*_*31*_ = 0.2, *b*_*32*_ = 0.5, *b*_*34*_ = 0.1, *b*_*35*_ = 0.2; *b*_*41*_ = 0.2, *b*_*42*_ = 0.1, *b*_*43*_ = 0.39, *b*_*45*_ = 0; *b*_*51*_ = 1.4, *b*_*52*_ = 0.7, *b*_*53*_ = 0.65, *b*_*54*_ = 0.
- Parameters of *f*_*i*_*(V*_*i*_*)* functions (mV): *V*_*1/2*_ = –30, *k*_*V1*_ = 8, *k*_*Vi*_ = 4 for *i* ≠ 1. Thus, these functions were identical for all neurons except the pre-I/I.
- Time constants (ms): τ_*hNaPmax*_ = 2,000, τ_*AD2*_ = 2,000, τ_*AD3*_ = 1,500, τ_*AD4*_ = 1,000, τ_*AD5*_ = 500.
- Adaptation parameters: *k*_*AD2*_ = 0.9, *k*_*AD3*_ = 0.9, *k*_*AD4*_ = 1.3, *k*_*AD5*_ = 1.7.
- External drives: *drive*_*i*_ = (0.21, 0.59, 0.73, 0.72, 0.3).
- Light stimuli: depending on the simulated experiments (see Results section), the light stimulation was applied either only to the inhibitory populations of the pre-BötC (early-I and post-I_pBC_), or only to inhibitory populations of BötC, or (for simulating the results of Alsahafi et al. [47]) to all populations of the pre-BötC (pre-I/I, early-I, and post-I_pBC_). The parameters (duration, intensity) of the applied light stimulation are indicated in the corresponding sections of the Results.

Simulations were performed using Matlab R2015b. Differential equations were solved using a variable order multistep differential equation solver ode15s available in Matlab.
